# Pan-cancer analyses of human nuclear receptors reveal transcriptome diversity and prognostic value across cancer types

**DOI:** 10.1038/s41598-020-58842-6

**Published:** 2020-02-05

**Authors:** Toshima Z. Parris

**Affiliations:** Department of Oncology, Institute of Clinical Sciences, Sahlgrenska Cancer Center, Sahlgrenska Academy at University of Gothenburg, Gothenburg, Sweden

**Keywords:** Bone cancer, Breast cancer, Cancer genomics, CNS cancer, Endocrine cancer, Eye cancer, Gastrointestinal cancer, Germ cell tumours, Gynaecological cancer, Haematological cancer, Head and neck cancer, Lung cancer, Mesothelioma, Oral cancer, Sarcoma, Skin cancer, Testicular cancer, Tumour biomarkers, Urological cancer, Biomarkers, Computational biology and bioinformatics

## Abstract

The human nuclear receptor (NR) superfamily comprises 48 ligand-dependent transcription factors that play regulatory roles in physiology and pathophysiology. In cancer, NRs have long served as predictors of disease stratification, treatment response, and clinical outcome. The Cancer Genome Atlas (TCGA) Pan-Cancer project provides a wealth of genetic data for a large number of human cancer types. Here, we examined NR transcriptional activity in 8,526 patient samples from 33 TCGA ‘Pan-Cancer’ diseases and 11 ‘Pan-Cancer’ organ systems using RNA sequencing data. The web-based Kaplan-Meier (KM) plotter tool was then used to evaluate the prognostic potential of NR gene expression in 21/33 cancer types. Although, most NRs were significantly underexpressed in cancer, NR expression (moderate to high expression levels) was predominantly restricted (46%) to specific tissues, particularly cancers representing gynecologic, urologic, and gastrointestinal ‘Pan-Cancer’ organ systems. Intriguingly, a relationship emerged between recurrent positive pairwise correlation of Class IV NRs in most cancers. NR expression was also revealed to play a profound effect on patient overall survival rates, with ≥5 prognostic NRs identified per cancer type. Taken together, these findings highlighted the complexity of NR transcriptional networks in cancer and identified novel therapeutic targets for specific cancer types.

## Introduction

Human nuclear receptors (NR) form a superfamily of 48 evolutionarily related transcription factors that rely on ligand binding (endogenous ligands: hormones, vitamins, and dietary lipids; exogenous ligands: pharmaceutical agents and toxins) and co-regulator recruitment to mediate the transcriptional activity of target genes^1–3^. NRs are typically comprised of five common domains: (i) nonconserved N-terminal A/B domain, (ii) highly conserved DNA-binding domain (DBD, C domain), (iii) flexible hinge between the DBD and LBD regions (D domain), (iv) moderately conserved ligand-binding domain (LBD, E domain), and (v) nonconserved C-terminal F domain^1^. Early phylogenetic studies further classified the NR superfamily into seven subfamilies or classes based on sequence similarity, including thyroid hormone receptors (class I), retinoid X receptors (class II), estrogen receptors (class III), nerve growth factors (class IV), steroidogenic factors (class V), germ cell nuclear factor (class VI), and class 0 NRs (*NR0B1* and *NR0B2*) that lack a DBD^4,5^. Though ligand binding predominantly occurs in the nucleus, a number of class III NRs (*ESR1* (*NR3A1*), *GR* (*NR3C1*), *MR* (*NR3C2*), *PR* (*NR3C3*) or *AR* (*NR3C4*)) bind to their respective ligands in the cytoplasm leading to subsequent NR translocation to the nucleus^6,7^. Despite similar structural architecture, differences in NR sequence homology (NR classes 0-VI), ligand binding (endocrine NRs, orphan NRs or adopted NRs), and NR-NR interaction (homo- and heterodimerization) ultimately result in tissue-specific responses^7–10^. NRs are therefore able to control a number of pivotal physiological processes (*e.g*. development, metabolism, reproduction, cell cycle, differentiation) and diseases (*e.g*. cancer, osteoporosis, diabetes, cardiovascular disease)^8,11^.

Consequently, about 16% of FDA-approved drugs currently target NRs, further highlighting the importance of NRs in human disease^3^. Due to their effect on various cancer-related processes (*e.g*. tumor initiation and therapeutic response), NRs have become attractive targets for anticancer drug development^10,12^. Tamoxifen, an estrogen receptor alpha (ERα) antagonist, was first introduced as a palliative agent for advanced breast cancer during the 1970s, but was later proven to be an effective adjuvant therapy for ERα-positive breast cancer patients^13^. Other pharmaceutical agents have since been FDA-approved for prostate cancer (androgen receptor (AR) antagonists), acute promyelocytic leukemia (retinoic acid receptor (RAR) agonists), AIDS-related Kaposi’s sarcoma (RAR and retinoid X receptor (RXR) agonists), and cutaneous T-cell lymphoma (RXR modulators), while others are currently in clinical trials (ER, AR, RAR, RXR, glucocorticoid receptor (GR), RAR-related orphan receptor (ROR), vitamin D receptor (VDR), peroxisome proliferator-activated receptor (PPAR), liver X receptor (LXR), and farnesoid X receptor (FXR))^10^. Other less well-known NRs, such as NR1I2 (PXR) and NR1I3 (CAR), have been shown to have an effect on the pharmacokinetics and pharmacodynamics of anticancer drugs^14,15^.

High-throughput sequencing technologies have been used to develop comprehensive insights into NR function and potential interplay between different NRs in cancer^8,16^. A pan-cancer study in six cancer types recently demonstrated that recurrent downregulation of NRs in cancer is only partially due to deletion or mutation^17^. Yet, our understanding of the impact global NR gene expression patterns have on patient clinical outcome is still limited in most cancer forms. Here, NR gene expression patterns were systematically mapped in relation to prognosis in 33 cancer types for 8,526 patients using genomic and clinical data from The Cancer Genome Atlas (TCGA), thereby pinpointing a number of interesting NR targets for future cancer drug development.

## Results

### RNA-seq analysis defines four main NR expression patterns in cancer

**Figure 1:**
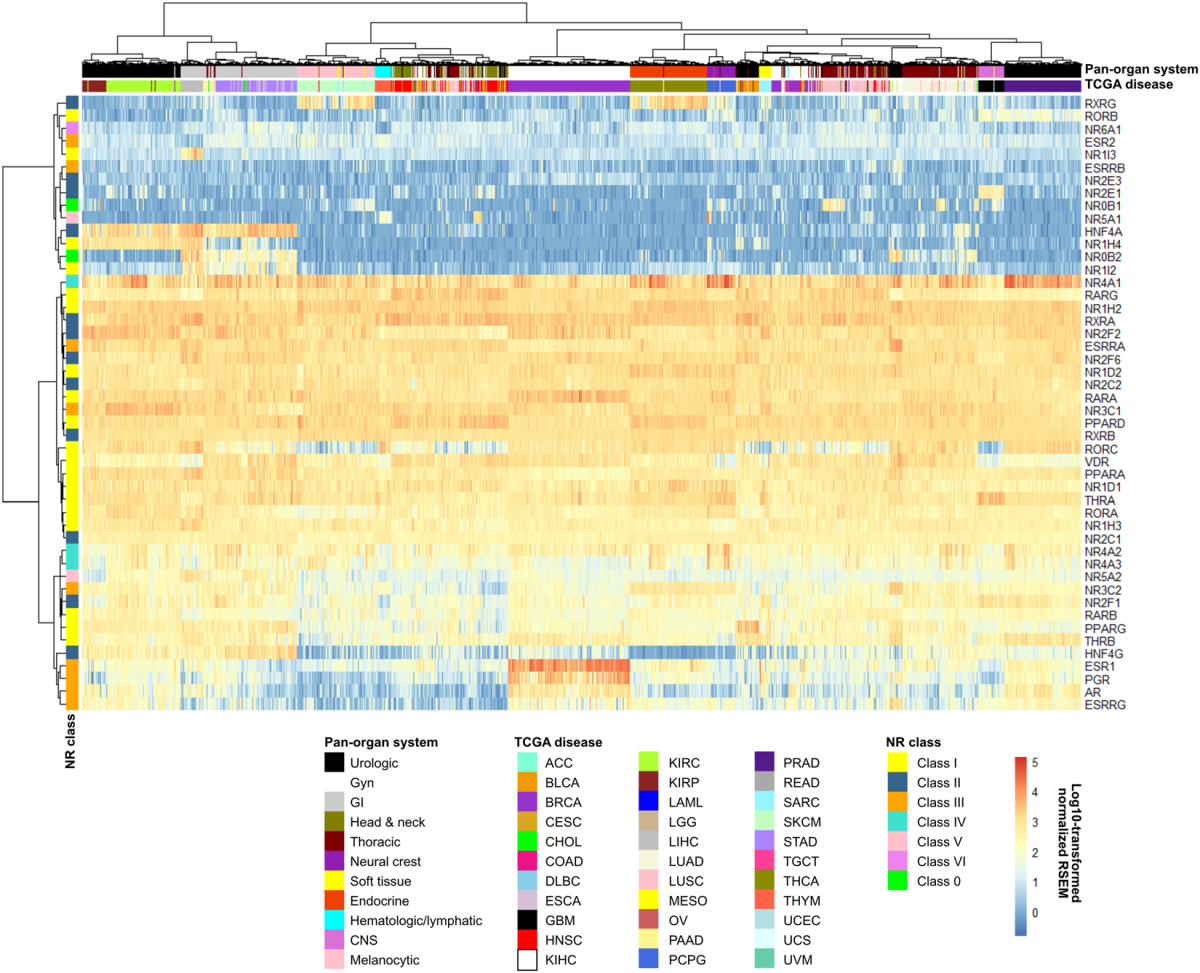
Human nuclear receptors display relatively similar expression patterns across ‘Pan-Cancer’ diseases. Heatmap depicting RNA-seq gene expression for 48 human NRs in 8,526 TCGA samples representing 33 ‘Pan-Cancer’ diseases. Hierarchical clustering was performed using the Manhattan distance metric and Ward’s minimum variance method (Ward.D2). Gene expression is shown in log10 normalized RSEM.

RNA-seq data for 8,526 TCGA patient samples were used to evaluate mRNA expression patterns for the 48 human NRs across 33 cancer types and 11 pan-organ groups (Tables 1–2). Evaluation of the genome-wide gene expression profiles revealed four main expression patterns in the different neoplastic tissues, *i.e*. absent (absent to low expression in 100% of tissues), restricted (expressed (defined as moderate to high expression levels) in <50% of tissues), widespread (expressed in >50%, but <100% of tissues), and ubiquitous (expressed in 100% of tissues). In total, five NRs (10%; *ESR2, ESRRB, NR2E3, NR6A1, RORB*) were not expressed in any tissue, whereas 22 NRs (46%; *AR*, *ESR1, ESRRG, HNF4A, HNF4G, NR0B1, NR0B2, NR1H4, NR1I2, NR1I3, NR2E1, NR2F1, NR3C2, NR4A3, NR5A1, NR5A2, PGR, PPARG, RARB, RORC, RXRG, THRB*) showed restricted expression patterns in specific ‘Pan-Cancer’ organ systems, *e.g*. gynecologic, endocrine, urologic, central nervous system, gastrointestinal, and thoracic (Supplementary Table 1). In contrast, 11 NRs (23%; *NR1D1, NR1H3, NR2F2, NR2F6, NR3C1, NR4A2, PPARA, RARG, RORA, THRA, VDR*) had widespread expression and 10 NRs (21%; *ESRRA, NR1D2, NR1H2, NR2C1, NR2C2, NR4A1, PPARD, RARA, RXRA, RXRB*) were ubiquitous. Interestingly, *ESRRG* (KIHC)*, NR0B1* (ACC)*, NR1I3* (LIHC)*, NR2E1* (GBM)*, NR5A1* (ACC) were only expressed in one neoplastic tissue (Fig. 1). Unsupervised hierarchical clustering of the expression profiles stratified the cohort fairly well by cancer type and pan-organ group. With the exception of three clusters of NRs representing NR classes I (cluster I: *RORC, VDR, PPARA, NR1D1, THRA, RORA, NR1H3*; cluster II: *RARB, PPARG, THRB*) and III (cluster III: *ESR1, PGR, AR, ESRRG*), NR class was not a good determinate of NR expression patterns in the different cancer types.

**Table 1:** TCGA cancer types and corresponding pan-cancer organ system.

Disease name and pan-organ system	Cohort	Number of samples	
		RNA-seq data^a^	KM plotter^b^
***Central nervous system (CNS)***			
Glioblastoma multiforme	GBM	166	0
Brain lower grade glioma	LGG	530	0
***Endocrine***			
Adrenocortical carcinoma	ACC	79	0
Thyroid carcinoma	THCA	496	502
***Gastrointestinal***			
Cholangiocarcinoma	CHOL	36	0
Colon adenocarcinoma	COAD	191	0
Esophageal adenocarcinoma	ESCA	185	80
Esophageal squamous cell carcinoma			81
Liver hepatocellular carcinoma	LIHC	147	371
Pancreatic adenocarcinoma	PAAD	56	177
Rectum adenocarcinoma	READ	72	165
Stomach adenocarcinoma	STAD	415	375
***Gynecologic***			
Breast invasive carcinoma	BRCA	1026	1090
Cervical and endocervical cancers	CESC	159	304
Ovarian serous cystadenocarcinoma	OV	265	374
Uterine corpus endometrial carcinoma	UCEC	369	543
***Head and neck***			
Head and Neck squamous cell carcinoma	HNSC	425	500
***Hematologic and lymphatic malignancies***			
Lymphoid neoplasm diffuse large B-cell lymphoma	DLBC	48	0
Acute myeloid leukemia	LAML	173	0
Thymoma	THYM	120	119
***Melanocytic***			
Skin cutaneous melanoma	SKCM	472	0
Uveal melanoma	UVM	80	0
***Neural crest-derived***			
Pheochromocytoma and Paraganglioma	PCPG	184	178
***Soft tissue***			
Sarcoma	SARC	105	259
Uterine carcinosarcoma	UCS	57	0
***Thoracic***			
Lung adenocarcinoma	LUAD	490	513
Lung squamous cell carcinoma	LUSC	482	501
Mesothelioma	MESO	87	0
***Urologic***			
Bladder urothelial carcinoma	BLCA	223	405
Kidney chromophobe	KIHC	66	0
Kidney renal clear cell carcinoma	KIRC	507	530
Kidney renal papillary cell carcinoma	KIRP	161	288
Prostate adenocarcinoma	PRAD	498	0
Testicular Germ Cell Tumors	TGCT	156	134
Total		8526	7489

^a^UNC RNASeqV2 level 3 expression (normalized RSEM) data were retrieved from Broad GDAC Firehose (https://gdac.broadinstitute.org/); ^b^Survival analysis was performed using the web-based Kaplan-Meier (KM) plotter tool, http://kmplot.com/analysis/index.php?p=service&cancer=pancancer_rnaseq.

**Table 2:** The 48 human nuclear receptors and associated ligands.

Gene symbol and full name		Gene abbreviation	NRNC Symbol	NR category^a^	Receptor	Ligand(s)^b^	Dimerization^c^	Associated cancer form(s)^a^
***Class I: thyroid hormone receptor-like***								
*THRA*	Thyroid hormone receptor-α	*THRα*	NR1A1	Endocrine	Thyroid hormone receptor	Thyroxine (T4), Triiodothyronine (T3)	Heterodimer/monomer	KIRC/KIRP
*THRB*	Thyroid hormone receptor-β	*THRβ*	NR1A2	Endocrine				
*RARA*	Retinoic acid receptor-α	*RARα*	NR1B1	Endocrine	Retinoic acid receptor	All-trans and 9-cis retinoic acid	Heterodimer	BRCA, COAD, SKCM
*RARB*	Retinoic acid receptor-β	*RARβ*	NR1B2	Endocrine				COAD, SKCM
*RARG*	Retinoic acid receptor-γ	*RARγ*	NR1B3	Endocrine				BRCA, COAD, SKCM
*PPARA*	Peroxisome proliferator-activated receptor-α	*PPARα*	NR1C1	Adopted	Peroxisome proliferator-activated receptor	Fatty acids	Heterodimer	KIRC/KIRP
*PPARD*	Peroxisome proliferator-activated receptor-β/δ	*PPARδ*	NR1C2	Adopted				KIRC/KIRP
*PPARG*	Peroxisome proliferator-activated receptor-γ	*PPARγ*	NR1C3	Adopted				HNSC, LIHC, COAD, LUAD/LUSC, KIRC/KIRP, SKCM
*NR1D1*	Rev-ErbAα	*REVERBα*	NR1D1	Adopted	Rev-ErbA	Heme	Monomer/homodimer	
*NR1D2*	Rev-ErbAα	*REVERBβ*	NR1D2	Adopted				
*RORA*	RAR-related orphan receptor-α	*RORα*	NR1F1	Adopted	RAR-related orphan receptor	Oxysterols	Monomer	HNSC
*RORB*	RAR-related orphan receptor-β	*RORβ*	NR1F2	Adopted		Cholesterol, cholesteryl sulphate		HNSC
*RORC*	RAR-related orphan receptor-γ	*RORγ*	NR1F3	Adopted		Retinoic acid		HNSC
*NR1H3*	Liver X receptor-α	*LXRα*	NR1H3	Adopted	Liver X receptor-like	Oxysterols		BRCA
*NR1H2*	Liver X receptor-β	*LXRβ*	NR1H2	Adopted		Oxysterols	Heterodimer	BRCA, SKCM
*NR1H4*	Farnesoid X receptor	*FXR*	NR1H4	Adopted		Bile acids	Heterodimer	LIHC, ESCA
*VDR*	Vitamin D receptor	*VDR*	NR1I1	Endocrine	Vitamin D receptor-like	Calcitriol (1',25' dihydroxy vitamin D3)	Heterodimer	HNSC, LIHC, COAD, BLCA, LUAD/LUSC
*NR1I2*	Pregnane X receptor	*PXR*	NR1I2	Adopted		Bile acids		
*NR1I3*	Constitutive androstane receptor	*CAR*	NR1I3	Adopted		Androstanol, androstenol		
***Class II: retinoid X receptor-like***								
*HNF4A*	Hepatocyte nuclear factor-4-α	*HNF4α*	NR2A1	Adopted	Hepatocyte nuclear factor-4	Fatty acids	Homodimer	COAD
*HNF4G*	Hepatocyte nuclear factor-4-γ	*HNF4γ*	NR2A2	Adopted				
*RXRA*	Retinoid X receptor-α	*RXRα*	NR2B1	Adopted	Retinoid X receptor	9-cis-retinoic acid	Heterodimer	BRCA, COAD, SKCM
*RXRB*	Retinoid X receptor-β	*RXRβ*	NR2B2	Adopted				BRCA, COAD, SKCM
*RXRG*	Retinoid X receptor-γ	*RXRγ*	NR2B3	Adopted				BRCA, COAD, LUAD/LUSC, SKCM
*NR2C1*	Testicular receptor 2	*TR2*	NR2C1	Orphan	Testicular receptor	All-trans retinoic acid	Homodimer/heterodimer	PRAD
*NR2C2*	Testicular receptor 4	*TR4*	NR2C2	Adopted				
*NR2E1*	Homologue of the Drosophila tailless gene	*TLX*	NR2E1	Orphan	TLX/PNR		Monomer/homodimer	PRAD
*NR2E3*	Photoreceptor cell-specific nuclear receptor	*PNR*	NR2E3	Orphan		Benzimidazoles		
*NR2F1*	Chicken ovalbumin upstream promoter-transcription factor I	*COUPTF1*	NR2F1	Orphan	COUP/EAR	Retinol/ATRA	Homodimer/heterodimer	
*NR2F2*	Chicken ovalbumin upstream promoter-transcription factor II	*COUPTF2*	NR2F2	Orphan				
*NR2F6*	V-erbA-related	*EAR2*	NR2F6	Orphan				
***Class III: estrogen receptor-like***								
*ESR1*	Estrogen receptor-α	*ERα*	NR3A1	Endocrine	Estrogen receptor	Estradiols	Homodimer	HNSC, BRCA, BLCA, OV
*ESR2*	Estrogen receptor-β	*ERβ*	NR3A2	Endocrine		Estradiols, 5α-androstane-3β, 17β-diol		COAD, OV
*ESRRA*	Estrogen-related receptor-α	*ERRα*	NR3B1	Adopted	Estrogen related receptor		Monomer/homodimer	OV
*ESRRB*	Estrogen-related receptor-β	*ERRβ*	NR3B2	Adopted				
*ESRRG*	Estrogen-related receptor-γ	*ERRγ*	NR3B3	Adopted				PRAD, OV
*NR3C1*	Glucocorticoid receptor	*GR*	NR3C1	Endocrine	3-Ketosteroid receptors	Cortisol (hydrocortisone)	Homodimer	BRCA, PRAD
*NR3C2*	Mineralocorticoid receptor	*MR*	NR3C2	Endocrine		Aldosterone		
*PGR*	Progesterone receptor	*PR*	NR3C3	Endocrine		Progesterone		BRCA, OV
*AR*	Androgen receptor	*AR*	NR3C4	Endocrine		Testosterone, dihydrotesterone		HNSC, BRCA, BLCA, PRAD, LUAD/LUSC
***Class IV: nerve growth factor IB-like***								
*NR4A1*	Nerve Growth factor IB	*NGFIB/NUR77*	NR4A1	Adopted	NGFIB/NURR1/NOR1		Monomer/homodimer/	
heterodimer	BLCA, KIRC/KIRP							
*NR4A2*	Nuclear receptor related 1	*NURR1*	NR4A2	Adopted				BLCA, PRAD
*NR4A3*	Neuron-derived orphan receptor 1	*NOR1*	NR4A3	Adopted				
***Class V: steroidogenic factor-like***								
*NR5A1*	NR5A1	*SF1*	NR5A1	Adopted	SF1/LRH1	Phospholipids	Monomer	
*NR5A2*	NR5A2	*LRH1*	NR5A2	Orphan				BRCA, COAD
***Class VI: germ cell nuclear factor-like***								
*NR6A1*	NR6A1	*GCNF*	NR6A1	Orphan	GCNF		Homodimer	
***Class 0: Miscellaneous***								
*NR0B1*	NR0B1	*DAX1*	NR0B1	Orphan	DAX/SHP		Heterodimer	PRAD
*NR0B2*	NR0B2	*SHP*	NR0B2	Orphan		CD437 Retinoids		LIHC, KIRC/KIRP

Data obtained from ^a^Dhiman VK *et al*., ^b^Zhao L *et al*., and ^c^Khorasanizadeh S *et al*.

### Differential gene expression reveals cancer-associated human NRs

**Figure 2:**
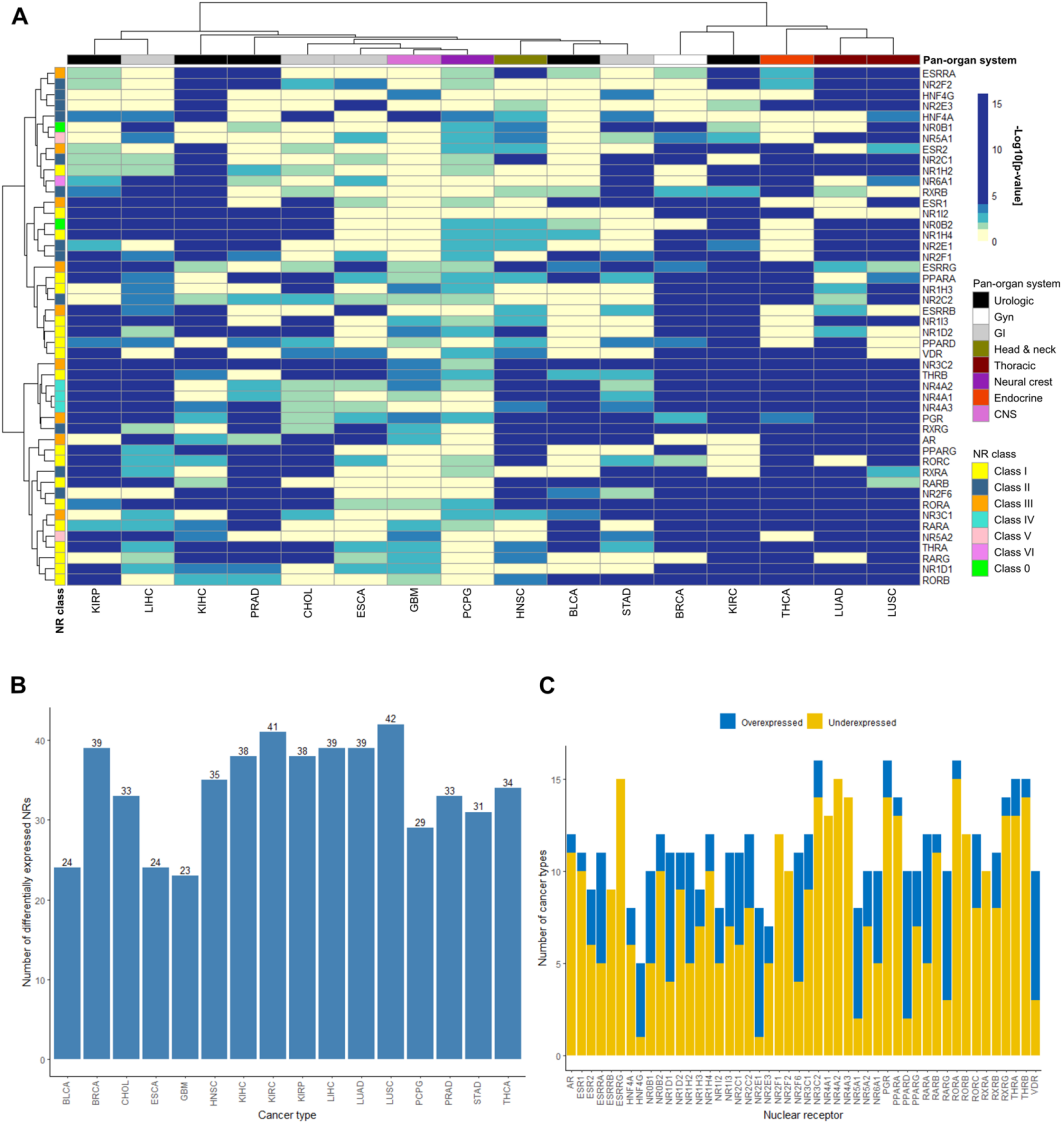
NRs are differentially expressed in normal and cancer tissue. (**A**) Heatmap of Benjamini-Hochberg adjusted p-values using the Wilcoxon test depicting differences in RNA-seq gene expression levels for 16 ‘Pan-Cancer’ forms and corresponding normal tissue. Hierarchical clustering was performed using the Manhattan distance metric and Ward’s minimum variance method (Ward.D2). Statistical significance is shown in −log10[adjusted p-value], where *P* < 0.05 corresponds to −log10[adjusted p-value] >1.3 (light green), *P* ≤ 0.01 corresponds to −log10[adjusted p-value] >2 (blue green), *P* ≤ 0.001 corresponds to −log10[adjusted p-value] >3 (green), and *P* ≤ 0.0001 corresponds to −log10[adjusted p-value] >4 (dark blue). **(B)** Bar chart depicting the number of differentially expressed NRs (cancer vs normal) that were identified per cancer type (corresponds to the number of green to blue colored rows in the heatmap). **(C)** Bar chart depicting the number of cancer types associated with over- (blue bars) and underexpression (yellow bars) of each NR in cancer compared with normal tissue (corresponds to the number of green to blue colored columns in the heatmap).

**Figure 3:**
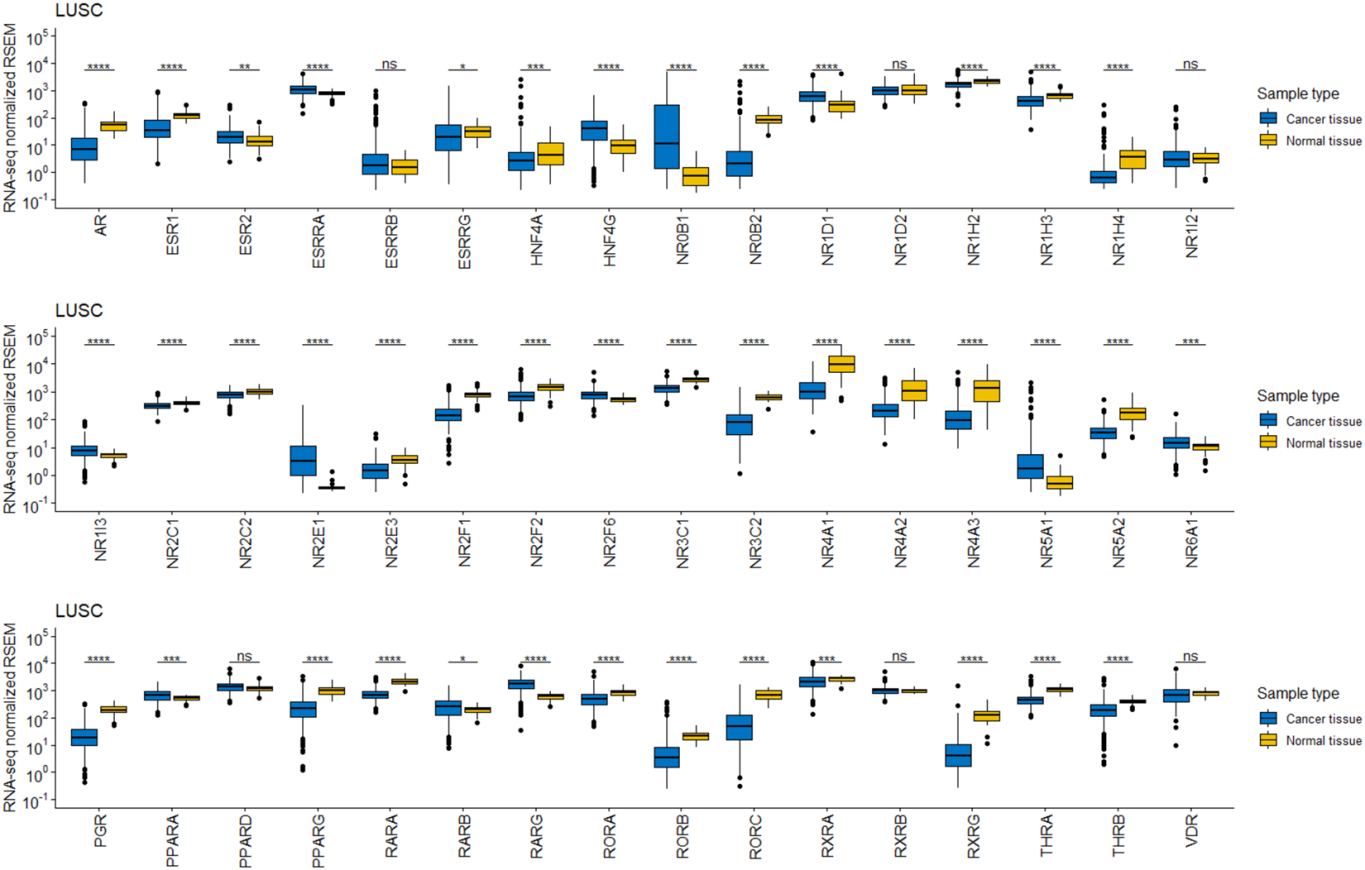
Strong association between NR gene expression and the LUSC cancer form. The highest number of differentially expressed NRs (42/48 NRs) was found in the LUSC cancer form. Box plots showing differences in NR gene expression levels between cancer and corresponding normal tissue for the LUSC cancer form. The Wilcoxon test was used to calculate statistical significance (Benjamini-Hochberg adjusted p-values). ns = not significant (*P* > 0.05); *P ≤ 0.05; **P ≤ 0.01; ***P ≤ 0.001; ****P ≤ 0.0001.

To identify cancer-related NRs, differential gene expression was assessed in cancer (n = 5,507) and corresponding normal tissue (n = 627) for 16 of the 33 pan-cancers with available gene expression data for normal samples. On average, 33.9 ± 1.60 ( ± SEM, range 23–42) NRs were differentially expressed per cancer type and 11.4 ± 0.40 (range 5–16) cancer types were associated with each NR (Fig. 2A–C). In addition, lower NR expression levels were prevalent in cancer compared with normal tissue. Interestingly, *NR3C2*, *PGR*, *RORA* were differentially expressed in all 16 cancer types, while *HNF4G* was differentially expressed in only 5/16 cancers (31.3%; Fig. 2C). The highest number of cancer-related NRs was found in LUSC (42 NRs, Fig. 3), KIRC (41 NRs), BRCA (39 NRs), LIHC (39 NRs), and LUAD (39 NRs), whereas only 23 differentially expressed NRs were significantly associated with GBM cancers.

### Pearson correlation demonstrates distinct patterns of NR co-expression in cancer

**Figure 4:**
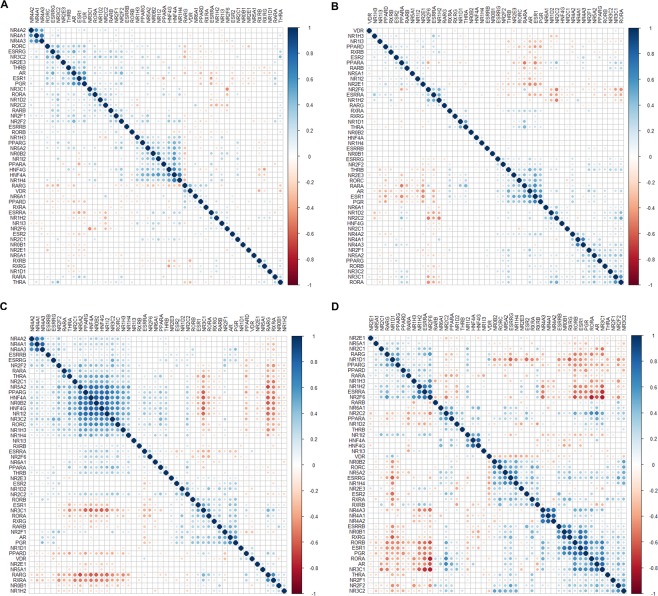
Pairwise Pearson correlation plots between NR gene expression in different ‘Pan-Cancer’ diseases. Correlation matrices for (**A**) the 21 ‘Pan-Cancer’ diseases, (**B**) BRCA, (**C**) ESCA, and (**D**) PAAD, with genes ordered using hierarchical clustering with the Ward’s minimum variance method (Ward.D2). Positive correlation coefficients are displayed in blue and negative correlation coefficients in red color. The color intensity and circle size are proportional to the correlation coefficients (*P* < 0.05), while correlation coefficients with *P* > 0.05 are blank.

Pairwise Pearson correlation was then used to assess co-expression of NRs in 21 of the 33 cancer types (Supplementary Figures). Examination of mRNA expression in all 21 cancer types revealed positive correlation between three NR gene clusters, namely 1) *NR4A1, NR4A2, NR4A3* (NR class IV), 2) *AR*, *ESR1*, *ESRRG*, *NR2E3*, *NR3C2*, *PGR*, *RORC*, *THRB* (NR class I/II/III), and 3) *HNF4A*, *HNF4G*, *NR0B2*, *NR1H3*, *NR1H4*, *NR1I2*, *NR5A2*, *PPARA*, *PPARG* (NR class 0/I/II/V; Fig. 4A). However, individual cancer types were also found to exhibit distinct NR co-expression patterns (Fig. 4B–D). NR expression patterns were generally shown to be weakly to moderately correlated (correlation coefficient values (*r*) between |0.2| and |0.6|) with the expression of other NRs in most neoplastic tissues. As expected, strong positive correlation (*r* > 0.6) was observed between the *ESR1, AR, PGR*, and *RARA* genes in BRCA. Intriguingly, evidence of NR crosstalk was found between NR class IV genes (*NR4A1*, *NR4A2*, *NR4A3*) in 20/21 cancer types (absent in SKCM). Only 6/21 cancer types (GI pan-organ system: ESCA, PAAD, STAD; Urologic pan-organ system: KIHC, PRAD; Hematologic/lymphatic pan-organ system: THYM) contained ≥20 strongly correlated (*r* > |0.6|) NR gene pairs (Supplementary Table 2). In total, 32 NR gene pairs were co-expressed in ESCA, several of which were comprised of the *HNF4A, HNF4G, NR0B2, NR1I2, NR3C2, NR4A1*, and *NR5A2* genes. Additionally, KIHC, PAAD, PRAD, and STAD cancers were found to be associated with a number of NR gene pairs containing at least one NR class III genes (estrogen receptor-like NRs, *e.g. AR, ESR1, ESRRA, ESRRB, ESRRG, NR3C1, NR3C2*, and *PGR*), whereas THYMs were strongly associated with NR class I genes (thyroid hormone receptor-like NRs, *e.g. PPAR, RAR, ROR* genes).

### The prognostic significance of NRs depends on the cancer type

**Figure 5:**
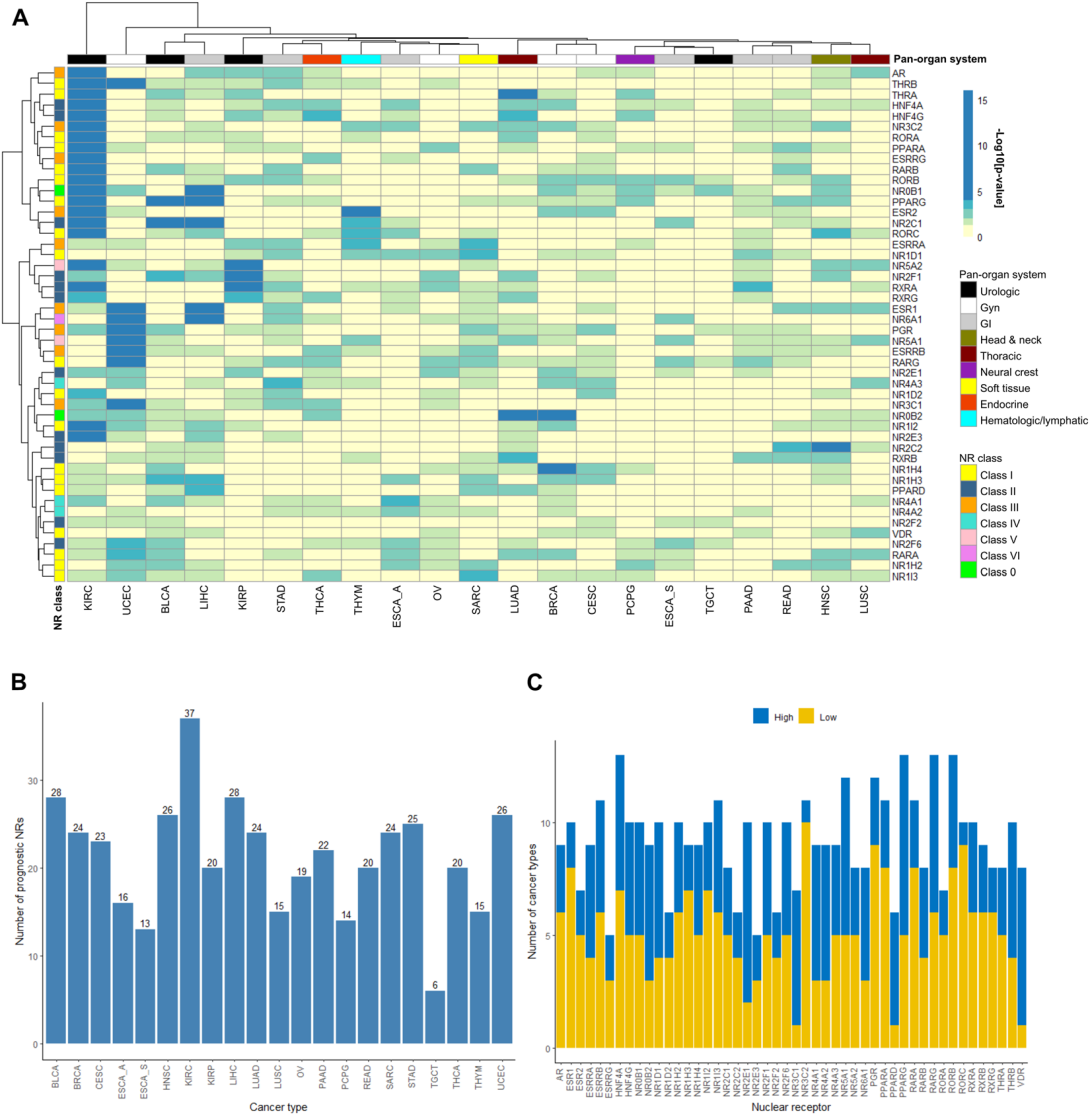
NRs are associated with clinical outcome for several ‘Pan-Cancer’ forms. (**A**) Heatmap of log-rank test p-values depicting the effect of NR gene expression on overall survival for 21 ‘Pan-Cancer’ forms. The ESCA ‘Pan-Cancer’ disease is shown as ESCA_A (esophageal adenocarcinoma) and ESCA_S (esophageal squamous cell carcinoma). Hierarchical clustering was performed using the Manhattan distance metric and Ward’s minimum variance method (Ward.D2). Statistical significance is shown in –log10[p-value], where *P* < 0.05 corresponds to −log10[p-value] >1.3 (light green), *P* ≤ 0.01 corresponds to −log10[p-value] >2 (blue green), *P* ≤ 0.001 corresponds to −log10[p-value] >3 (green), and *P* ≤ 0.0001 corresponds to −log10[p-value] >4 (dark blue). (**B**) Bar chart depicting the number of identified prognostic NRs per cancer type (corresponds to the number of green to blue colored rows in the heatmap). **(C)** Bar chart depicting the number of cancer types associated with high (blue bars) and low expression (yellow bars) for each prognostic NR (corresponds to the number of green to blue colored columns in the heatmap).

**Figure 6:**
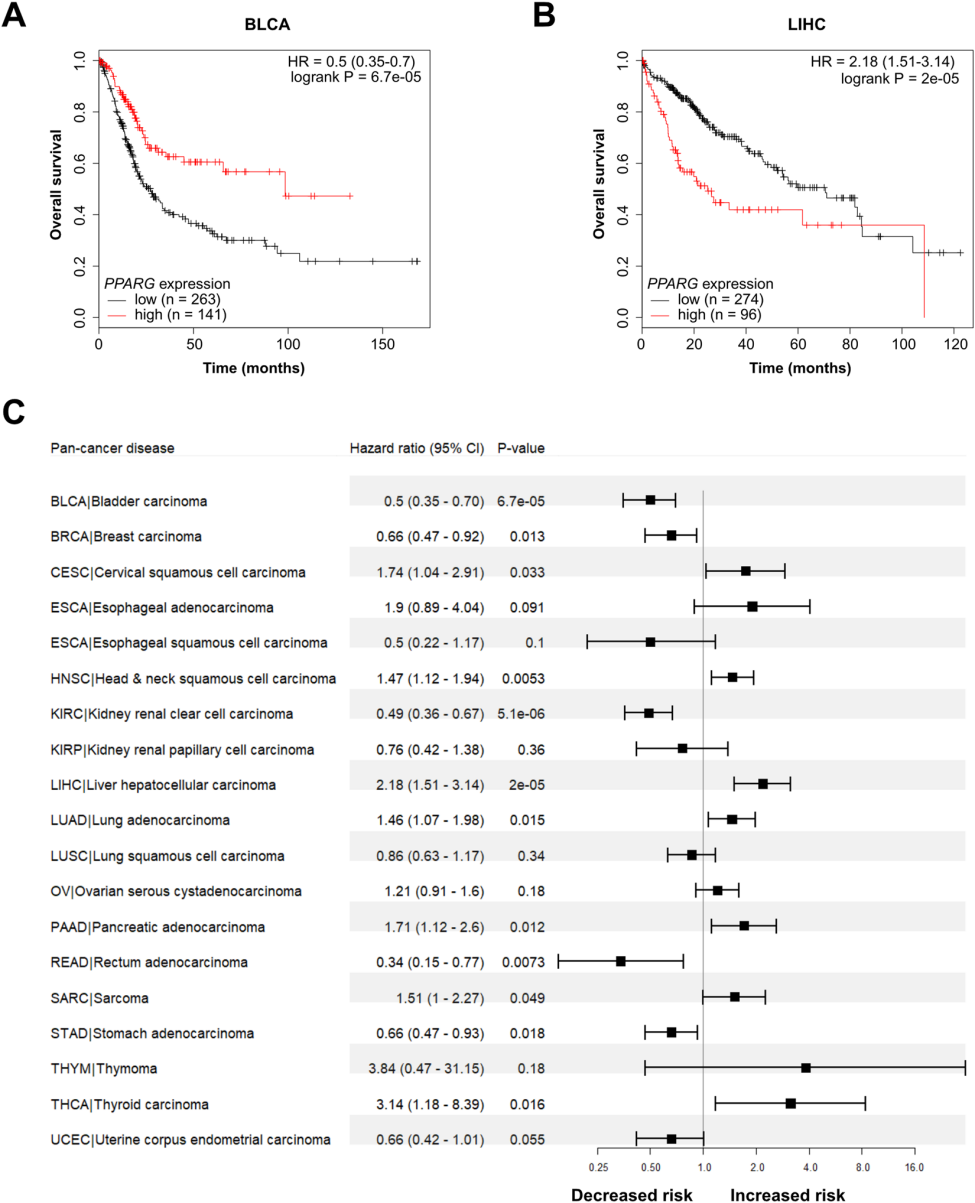
Gene expression of the *PPARG* nuclear receptor is significantly associated with overall survival in cancer. (**A**,**B**) Kaplan–Meier analysis of *PPARG* expression in the BLCA and LIHC cohorts. Estimates of the probability of overall survival according to quantile expression (low or high expression). P-values, hazard ratios (HR), and 95% confidence intervals (95% CI) were calculated using the log-rank test and Cox proportional hazards regression, respectively. The x-axes depict months after initial diagnosis and the y-axes depict overall survival. (**C**) Forest plots illustrating univariate Cox regression analysis of the prognostic impact of *PPARG* expression on overall survival in 19 ‘Pan-Cancer’ forms. The x-axis is in log scale. HR <1 depicts the association between high *PPARG* expression and decreased risk, whereas HR >1 illustrates the association between high *PPARG* expression and increased risk.

Furthermore, the prognostic potential of NR expression was examined in 21 pan-cancers using the web-based Kaplan-Meier (KM) plotter tool with dichotomized gene expression (high and low expression) and overall survival times (Supplementary Figures). Although a number of ‘Pan-Cancer’ diseases (BRCA/CESC and PAAD/READ) and NRs (NR class I: *THRA*/*THRB*, *RORA*/*PPARA*, *RARB*/*RORB*, *NR1H3*/*NR1H4*/*PPARD*, and *RARA*/*NR1H2*/*NR1I3;* NR class II: *HNF2A*/*HNF4G*, *NR2F1*/*RXRA*/*RXRG*, and *NR2E3*/*NR2C2*/*RXRB*; NR class IV: *NR4A1*/*NR4A2*) belonging to the same groups clustered together, hierarchical clustering of the log-rank test p-values showed no clear correlation between prognostic potential and pan-organ system or NR class (Fig. 5A). On average, 21.2 ± 1.5 (±SEM, range 6–37) NRs were significantly associated with overall survival per cancer type and 9.2 ± 0.3 (range 5–13) cancer types were associated with each prognostic NR (Fig. 5B). Although NRs were generally found to be underexpressed in cancer compared with corresponding normal tissue, both high and low NR expression correlated with adverse clinical outcome (Fig. 5C). Consequently, the prognostic significance of an individual NR frequently differed for cancer types in the same ‘Pan-Cancer’ organ system. *NR2E1* was the only NR to demonstrate an association between similar expression patterns and shorter overall survival rates in all cancer types within a ‘Pan-Cancer’ organ system (high *NR2E1* expression in BRCA, CESC, OV, and UCEC among gynecologic pan-cancers). In contrast, the prognostic potential of the remaining 47 NRs was frequently found to be connected with diverse expression patterns in different cancer types within a ‘Pan-Cancer’ organ system and NR class. For example, high *PPARG* expression was shown to be associated with decreased risk for BRCA and increased risk for CESC in the gynecologic organ system, and decreased risk for READ/STAD and increased risk for LIHC/PAAD cancers in the GI organ system. Furthermore, high *PPARG* expression was found to have a protective effect in five cancer types, *e.g*. BLCA (HR = 0.5, 95% CI 0.35–0.7, *P* = 6.7e-05; Fig. 6A), and an adverse effect in seven cancer types, *e.g*. LIHC (HR = 2.18, 95% CI 1.51–3.14, *P* = 2e-05; Fig. 6B). The effect of *PPARG* expression on patient clinical outcome thereby depended on the cancer type (Fig. 6C).

## Discussion

Although the spatial (expression in different tissues) and temporal (circadian regulation) effects of NR function have been studied extensively in normal mouse tissues, no large-scale studies have currently been conducted in human cancers^17–19^. Therefore, publicly accessible TCGA data containing genome-wide molecular datasets and matching clinical information offers a unique opportunity to examine the relationship between NR expression and prognosis in a range of cancer forms. This comprehensive analysis of global NR gene expression patterns in 33 TCGA cancer types provides a detailed description of NR expression and potential co-expression in specific neoplastic tissues and pan-cancer organ groups. Here, the vast majority of NRs were shown to either be expressed in specific neoplastic tissues (restricted), most tissues (widespread), or all tissues (ubiquitous). Consistent with a previous report, NR expression was generally down-regulated in cancer compared with corresponding normal tissue^17^. However, this is the first report, to evaluate the clinical utility of the NR superfamily in cancer using survival analysis.

Crystollographic studies have improved our knowledge of how one or more NR polypeptides form dimers (mono-, homo- or heterodimeric NRs) that eventually bind to DNA response elements (DNA direct repeats, palindromic repeats or monomeric sites) in the nucleus^9^. Intriguingly, gene expression analysis showed that approximately 20% of NRs (*ESRRA*, *NR1D2*, *NR1H2*, *NR2C1*, *NR2C2*, *NR4A1*, *PPARD*, *RARA*, *RXRA*, *RXRB*) were ubiquitously expressed in all 33 pan-cancers. Furthermore, pairwise Pearson correlation for 21/33 cancer types revealed recurrent correlation between the expression patterns for multiple class I-III NRs and retinoid X receptors (RXRs), as well as, strong positive correlation between class IV NRs (*NR4A1*, *NR4A2*, *NR4A3*) in 20/21 pan-cancers. Although less prevalent, negative correlation was also observed, *e.g. RARA* and *PPARA*, *ESR1* and *PPARA*, *NR2E1* and *RARA*/*AR*/*ESR1*/*PGR*, *NR2C2* and *NR2F6*/*NR1H2* in BRCA, which is in line with previous reports^20^. Correlation between expression patterns for RXR genes and other NRs is not surprising since RXRs are common heterodimer partners with class I/II NRs (*e.g*. RAR, TR, VDR, LXR, PPAR, FXR, PXR, CAR)^7,9^. Indeed, class IV NRs have been previously associated with urologic malignancies such as bladder urothelial carcinoma, kidney renal carcinoma, and prostate adenocarcinoma, but this is the first report of widespread coordinated expression between these NRs in cancer^8^.

Survival analysis demonstrated that the prognostic potential of NR expression is predominantly dependent on cancer type, rather than on NR class. Each cancer type and NR were shown to be associated with ≥6 prognostic NRs and ≥5 different cancers, respectively. However, the expression levels (low or high expression) of individual prognostic NRs frequently differed between cancer types. For example, high *PPARG* expression correlated with decreased mortality risk for five cancer types (BLCA, BRCA, KIRC, READ, and STAD) and increased risk for eight cancer types (CESC, HNSC, LIHC, LUAD, PAAD, PCPG, SARC, THCA). Surprisingly, *NR2E1* was the only NR to display similar expression levels (high expression) in association with overall survival in all cancer types (BRCA, CESC, OV, and UCEC) within a pan-cancer organ system (gynecologic pan-cancers).

In summary, this integrative pan-cancer analysis provides a detailed overview of the effects of NR expression on clinical outcome, thereby highlighting the importance of NRs in cancer. This work confirmed previously identified relationships between individual NRs and specific cancer types and revealed novel clinically relevant NRs. Taken together, these findings may therefore prompt a reevaluation of certain NRs as potential actionable targets for various cancer forms.

## Methods

### Patient cohorts and data acquisition

Genomic and clinical data for 33 cancer types from The Cancer Genome Atlas (TCGA) consortium were retrieved from Broad GDAC Firehose (https://gdac.broadinstitute.org/). The patient cohorts were further stratified into 11 pan-organ systems (central nervous system (CNS), endocrine, gastrointestinal, gynecologic, head and neck, hematologic and lymphatic malignancies, melanocytic, neural-crest derived, soft tissue, thoracic, urologic).

UNC RNASeqV2 level 3 expression (normalized RSEM) data for the 48 human NRs (*AR, ESR1, ESR2, ESRRA, ESRRB, ESRRG, HNF4A, HNF4G, NR0B1, NR0B2, NR1D1, NR1D2, NR1H2, NR1H3, NR1H4, NR1I2, NR1I3, NR2C1, NR2C2, NR2E1, NR2E3, NR2F1, NR2F2, NR2F6, NR3C1, NR3C2, NR4A1, NR4A2, NR4A3, NR5A1, NR5A2, NR6A1, PGR, PPARA, PPARD, PPARG, RARA, RARB, RARG, RORA, RORB, RORC, RXRA, RXRB, RXRG, THRA, THRB, VDR;* Table 2) were retrieved from Broad GDAC Firehose for 8,526 TCGA tumor specimens and 627 normal specimens. The prognostic significance of the 48 NRs was assessed using the web-based Kaplan-Meier (KM) plotter tool (http://kmplot.com/analysis/index.php?p = service&cancer = pancancer_rnaseq) with 7,489 TCGA RNA-seq datasets representing 21 different ‘Pan-Cancer’ diseases (the ESCA cohort was stratified into ESCA_A (esophageal adenocarcinoma) and ESCA_S (esophageal squamous cell carcinoma)). The patient cohorts are described in detail in Table 1^21^.

### Statistical analysis

Statistical analyses were performed using a 0.05 p-value cutoff in R/Bioconductor (version 3.6.0). All p-values are two-sided. The distribution of NR gene expression levels was evaluated in each cancer type by calculating quantile expression (Q1–Q4) using log10-transformed RNA-seq data. Expression levels were then classified as not expressed (Q1 (0–25%: -Inf to 0.98) were defined as absent and Q2 (25–50%: 0.98 to 2.32) as low expression) or expressed (Q3 (50–75%: 2.32 to 2.93) as moderate and Q4 (75–100%: 2.93 to 5.13) as high expression). The frequency of NR expression in a given cancer type was defined as absent (absent to low expression in 100% of tissues), restricted (expressed in <50% of tissues), widespread (expressed in >50%, but <100% of tissues), and ubiquitous (expressed in 100% of tissues), as described elsewhere^18^. The KM plotter tool first dichotomized gene expression into high and low expression using median expression as a cut-off and then constructed Kaplan-Meier plots by calculating univariate Cox proportional hazard models for the 48 genes using overall survival (OS) and log-rank test (Supplementary Figures). Hierarchical clustering of the log10-tranformed RNA-seq data and -log10-tranformed p-values (survival analysis) was performed with the pheatmap R package (version 1.0.12)^22^ using the Manhattan distance metric and Ward’s minimum variance method (Ward.D2). Box plots were constructed using the ggpubr (version 0.2.1.999)^23^ and rstatix (version 0.1.1.999)^24^ R packages to compare gene expression levels between cancer and normal samples with the Wilcoxon test and Benjamini-Hochberg adjusted p-values. Cancer types with no available normal samples (ACC, CESC, COAD, DLBC, LAML, LGG, MESO, OV, PAAD, READ, SARC, SKCM, TGCT, THYM, UCEC, UCS, UVM) were excluded from the analysis. The pairwise Pearson’s correlation coefficient (*r*) was calculated per gene pair using the basic stats R package to determine the level of co-expression. Gene expression correlation matrices were visualized using the corrplot R package with Ward D2 hierarchical clustering and *P* < 0.05 (95% CI) (version 0.84)^25^. Forest plots were used to display hazard ratios (HR) for the effect of gene expression on overall survival with the forestplot R package (version 1.9)^26^.

## Supplementary information

Supplementary Information.

Supplementary Information.

## References

[1] Mangelsdorf DJ (1995). The nuclear receptor superfamily: the second decade. Cell..

[2] Millard CJ, Watson PJ, Fairall L, Schwabe JW (2013). An evolving understanding of nuclear receptor coregulator proteins. Journal of molecular endocrinology.

[3] Santos R (2017). A comprehensive map of molecular drug targets. Nature reviews. Drug discovery.

[4] Committee NRN (1999). A unified nomenclature system for the nuclear receptor superfamily. Cell..

[5] Laudet V (1997). Evolution of the nuclear receptor superfamily: early diversification from an ancestral orphan receptor. Journal of molecular endocrinology.

[6] Giovannelli P (2011). Targeting rapid action of sex steroid receptors in breast and prostate cancers. Front Biosci (Landmark Ed).

[7] Khorasanizadeh S, Rastinejad F (2016). Visualizing the Architectures and Interactions of Nuclear Receptors. Endocrinology.

[8] Dhiman VK, Bolt MJ, White KP (2018). Nuclear receptors in cancer - uncovering new and evolving roles through genomic analysis. Nature reviews. Genetics.

[9] Rastinejad F, Huang P, Chandra V, Khorasanizadeh S (2013). Understanding nuclear receptor form and function using structural biology. Journal of molecular endocrinology.

[10] Zhao, L., Zhou, S. & Gustafsson, J. A. Nuclear receptors: recent drug discovery for cancer therapies. *Endocr Rev*, 10.1210/er.2018-00222 (2019). 10.1210/er.2018-00222 30869771

[11] Escriva, H., Delaunay, F. & Laudet, V. Ligand binding and nuclear receptor evolution. *Bioessays* **22**, 717–727, 10.1002/1521-1878(200008)22:8<717::Aid-bies5>3.0.Co;2-i (2000). 10.1002/1521-1878(200008)22:8<717::AID-BIES5>3.0.CO;2-I 10918302

[12] Sherman MH, Downes M, Evans RM (2012). Nuclear receptors as modulators of the tumor microenvironment. Cancer prevention research (Philadelphia, Pa.).

[13] Jordan VC (2014). Tamoxifen as the first targeted long-term adjuvant therapy for breast cancer. Endocrine-related cancer.

[14] Cecchin E, De Mattia E, Toffoli G (2016). Nuclear receptors and drug metabolism for the personalization of cancer therapy. Expert opinion on drug metabolism & toxicology.

[15] Harmsen S, Meijerman I, Beijnen JH, Schellens JH (2007). The role of nuclear receptors in pharmacokinetic drug-drug interactions in oncology. Cancer treatment reviews.

[16] Kittler R (2013). A comprehensive nuclear receptor network for breast cancer cells. Cell reports.

[17] Long, M. D. & Campbell, M. J. Pan-cancer analyses of the nuclear receptor superfamily. *Nuclear receptor research* **2**, 10.11131/2015/101182 (2015). 10.11131/2015/101182 PMC4869537 27200367

[18] Bookout AL (2006). Anatomical profiling of nuclear receptor expression reveals a hierarchical transcriptional network. Cell.

[19] Yang X (2006). Nuclear receptor expression links the circadian clock to metabolism. Cell.

[20] Lin ML (2015). Expression profiling of nuclear receptors in breast cancer identifies TLX as a mediator of growth and invasion in triple-negative breast cancer. Oncotarget.

[21] Nagy A, Lanczky A, Menyhart O, Gyorffy B (2018). Validation of miRNA prognostic power in hepatocellular carcinoma using expression data of independent datasets. Scientific Reports.

[22] Kolde, R. R package “pheatmap”: Pretty Heatmaps (2019).

[23] Kassambara, A. R package “ggpubr”: ‘ggplot2’ Based Publication Ready Plots. (2019).

[24] Kassambara, A. R package “rstatix”: Pipe-Friendly Framework for Basic Statistical Tests. (2019).

[25] Wei, T. & Simko, V. R package “corrplot”: Visualization of a Correlation Matrix. (2017).

[26] Gordon, M. & Lumley, T. R package “forestplot”: Advanced Forest Plot Using ‘grid’ Graphics. (2019).

