# Supplementary material for: Pan-cancer analyses of human nuclear receptors reveal transcriptome diversity and prognostic value across cancer types
Source: Sci Rep. 2020 Feb 5;10:1873. doi: 10.1038/s41598-020-58842-6 (PMC7002682; doi:10.1038/s41598-020-58842-6)
Supplement: Supplementary file 2 — Supplementary Information. [file 41598_2020_58842_MOESM2_ESM.pdf]

All cancers

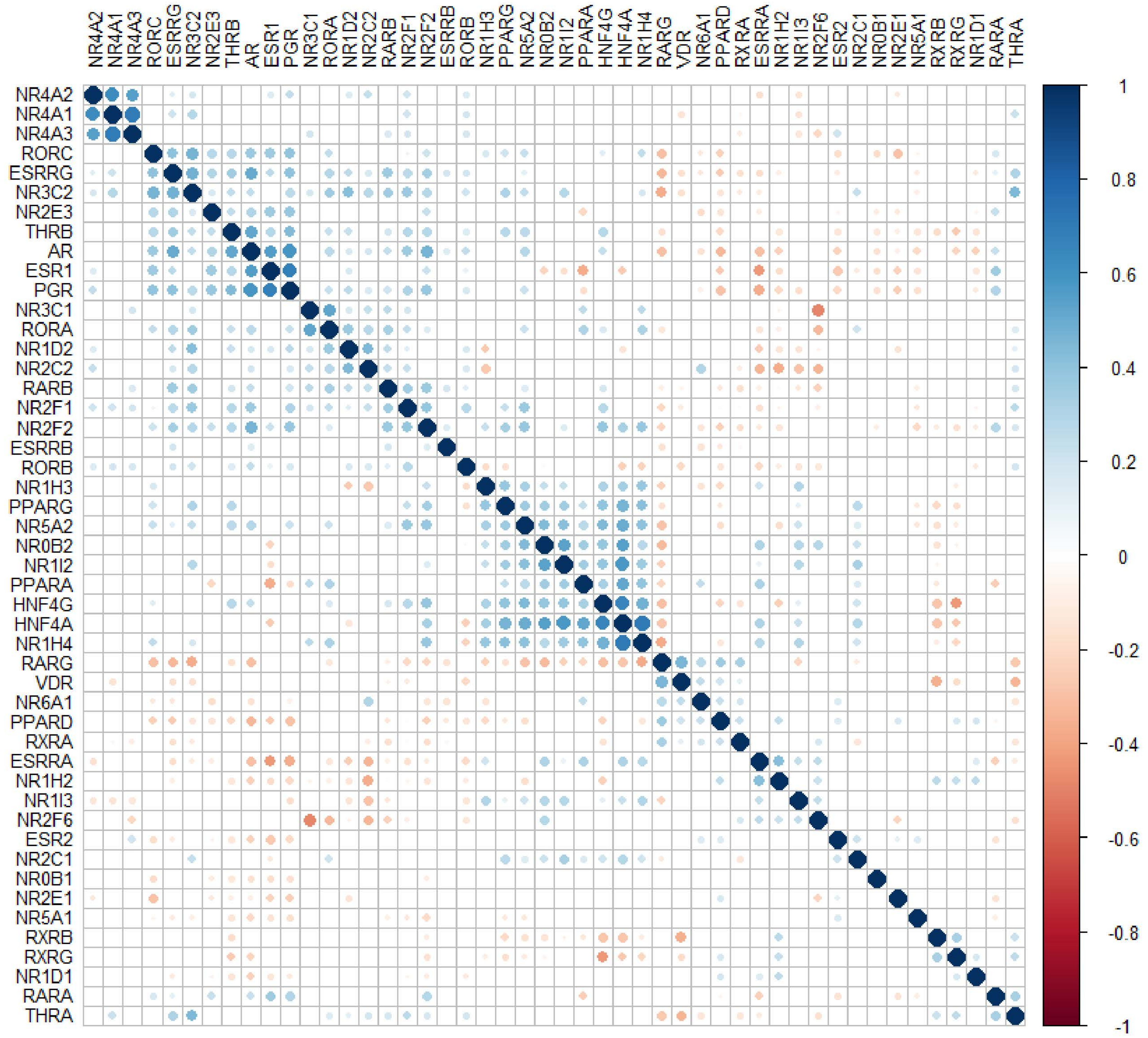

NR4A2
NR4A1
NR4A3
RORC
ESRRG
NR3C2
NR2E3
THRB
AR
ESR1
PGR
NR3C1
RORA
NR1D2
NR2C2
RARB
NR2F1
NR2F2
ESRRB
RORB
NR1H3
PPARG
NR5A2
NR0B2
NR1I2
PPARA
HNF4G
HNF4A
NR1H4
RARG
VDR
NR6A1
PPARD
RXRA
ESRRA
NR1H2
NR1I3
NR2F6
ESR2
NR2C1
NR0B1
NR2E1
NR5A1
RXRB
RXRG
NR1D1
RARA
THRA
1
0.8
0.6
0.4
0.2
0
-0.2
-0.4
-0.6
-0.8
-1

BLCA

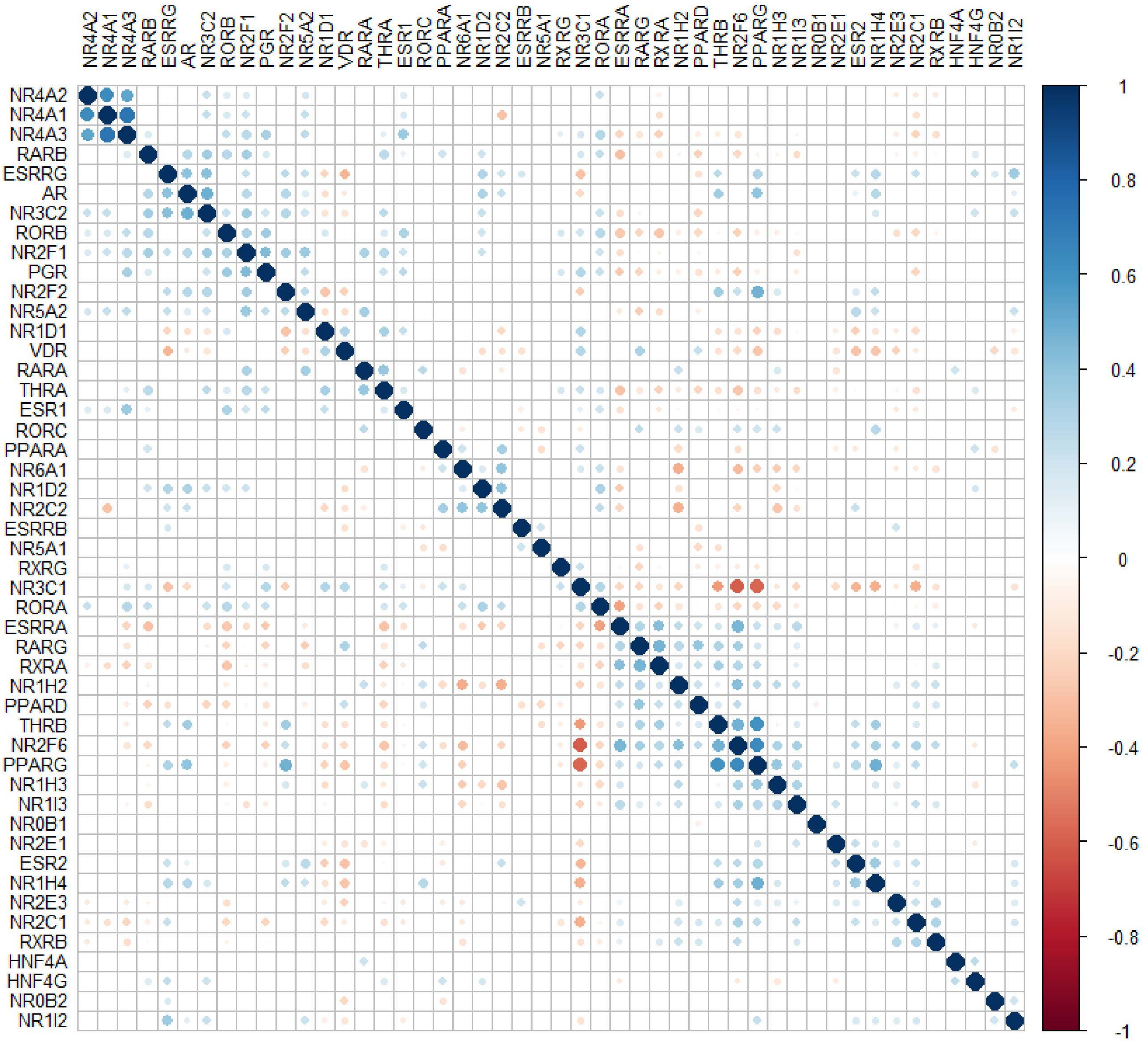

NR4A2
NR4A1
NR4A3
RARB
ESRRG
AR
NR3C2
RORB
NR2F1
PGR
NR2F2
NR5A2
NR1D1
VDR
RARA
THRA
ESR1
RORC
PPARA
NR6A1
NR1D2
NR2C2
ESRRB
NR5A1
RXRG
NR3C1
RORA
ESRRA
RARG
RXRA
NR1H2
PPARD
THRB
NR2F6
PPARG
NR1H3
NR1I3
NR0B1
NR2E1
ESR2
NR1H4
NR2E3
NR2C1
RXRB
HNF4A
HNF4G
NR0B2
NR1I2
1
0.8
0.6
0.4
0.2
0
-0.2
-0.4
-0.6
-0.8
-1

BRCA

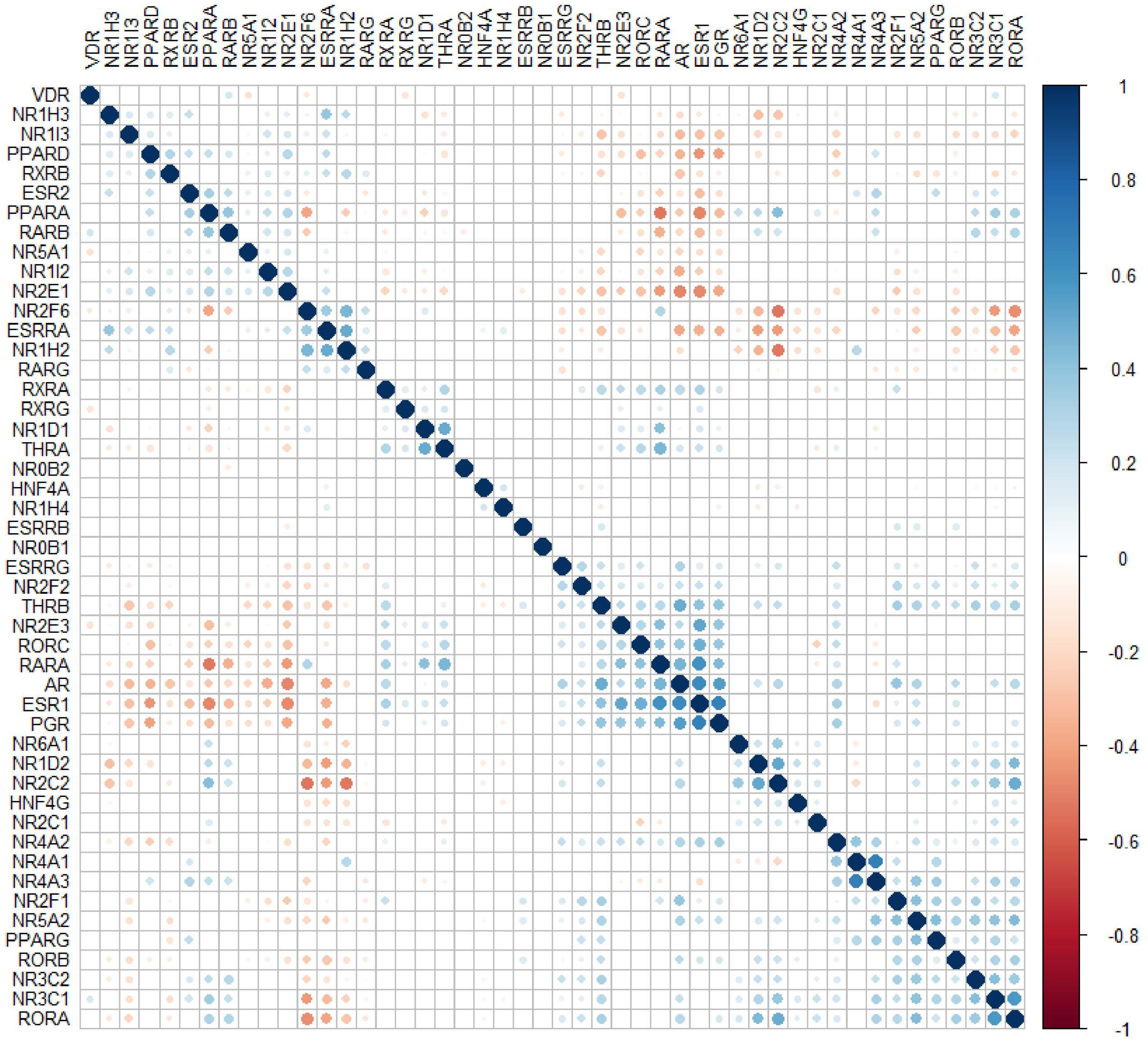
VDR
NR1H3
NR1I3
PPARD
RXRB
ESR2
PPARA
RARB
NR5A1
NR1I2
NR2E1
NR2F6
ESRRA
NR1H2
RARG
RXRA
RXRG
NR1D1
THRA
NR0B2
HNF4A
NR1H4
ESRRB
NR0B1
ESRRG
NR2F2
THRB
NR2E3
RORC
RARA
AR
ESR1
PGR
NR6A1
NR1D2
NR2C2
HNF4G
NR2C1
NR4A2
NR4A1
NR4A3
NR2F1
NR5A2
PPARG
RORB
NR3C2
NR3C1
RORA
1
0.8
0.6
0.4
0.2
0
-0.2
-0.4
-0.6
-0.8
-1

CESC

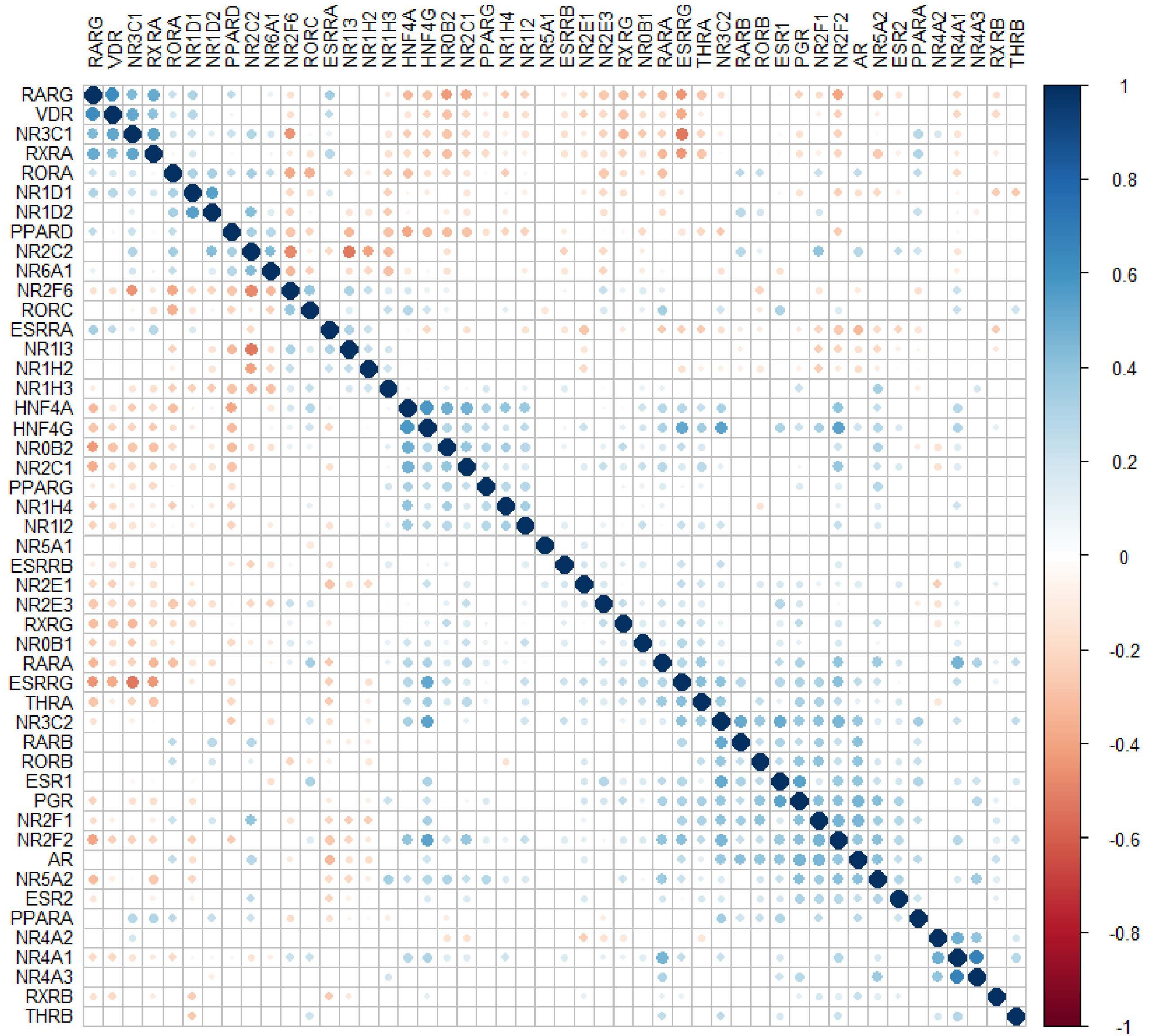

RARG
VDR
NR3C1
RXRA
RORA
NR1D1
NR1D2
PPARD
NR2C2
NR6A1
NR2F6
RORC
ESRRA
NR1I3
NR1H2
NR1H3
HNF4A
HNF4G
NR0B2
NR2C1
PPARG
NR1H4
NR1I2
NR5A1
ESRRB
NR2E1
NR2E3
RXRG
NR0B1
RARA
ESRRG
THRA
NR3C2
RARB
RORB
ESR1
PGR
NR2F1
NR2F2
AR
NR5A2
ESR2
PPARA
NR4A2
NR4A1
NR4A3
RXRB
THRB
1
0.8
0.6
0.4
0.2
0
-0.2
-0.4
-0.6
-0.8
-1

CHOL

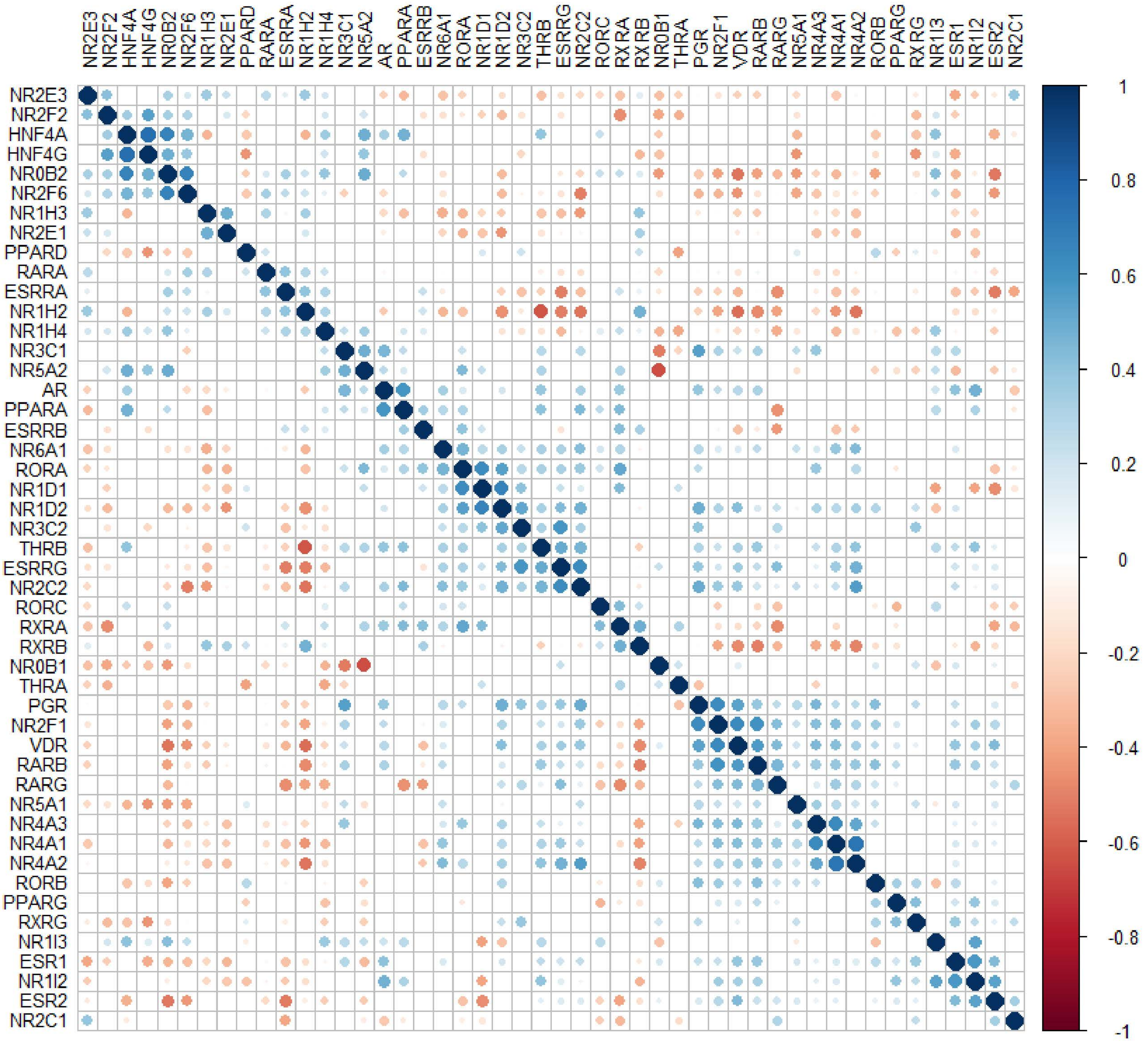

NR2E3
NR2F2
HNF4A
HNF4G
NR0B2
NR2F6
NR1H3
NR2E1
PPARD
RARA
ESRRA
NR1H2
NR1H4
NR3C1
NR5A2
AR
PPARA
ESRRB
NR6A1
RORA
NR1D1
NR1D2
NR3C2
THRB
ESRRG
NR2C2
RORC
RXRA
RXRB
NR0B1
THRA
PGR
NR2F1
VDR
RARB
RARG
NR5A1
NR4A3
NR4A1
NR4A2
RORB
PPARG
RXRG
NR1I3
ESR1
NR1I2
ESR2
NR2C1
1
0.8
0.6
0.4
0.2
0
-0.2
-0.4
-0.6
-0.8
-1

ESCA

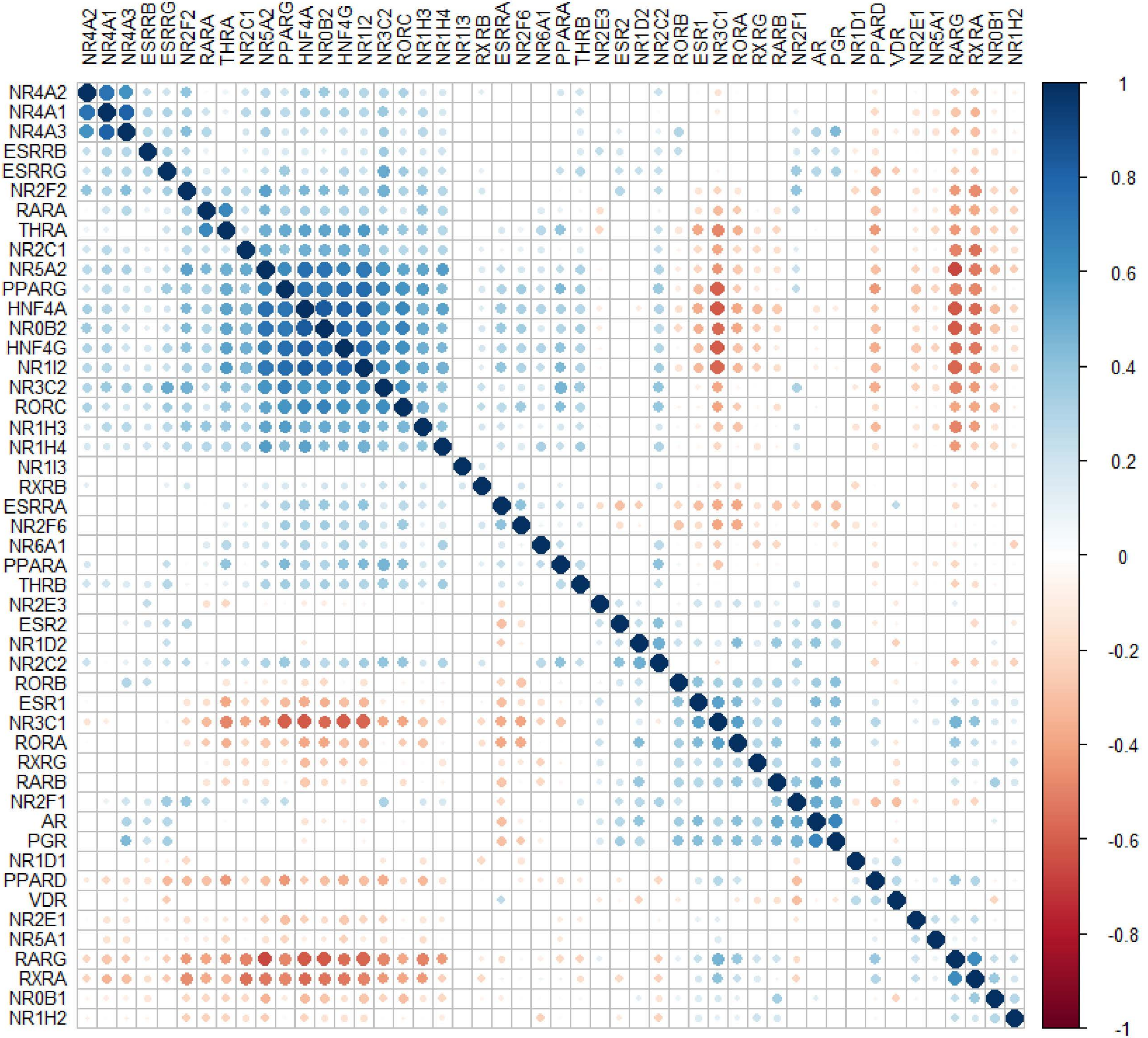
NR4A2
NR4A1
NR4A3
ESRRB
ESRRG
NR2F2
RARA
THRA
NR2C1
NR5A2
PPARG
HNF4A
NR0B2
HNF4G
NR1I2
NR3C2
RORC
NR1H3
NR1H4
NR1I3
RXRB
ESRRA
NR2F6
NR6A1
PPARA
THRB
NR2E3
ESR2
NR1D2
NR2C2
RORB
ESR1
NR3C1
RORA
RXRG
RARB
NR2F1
AR
PGR
NR1D1
PPARD
VDR
NR2E1
NR5A1
RARG
RXRA
NR0B1
NR1H2
1
0.8
0.6
0.4
0.2
0
-0.2
-0.4
-0.6
-0.8
-1

GBM

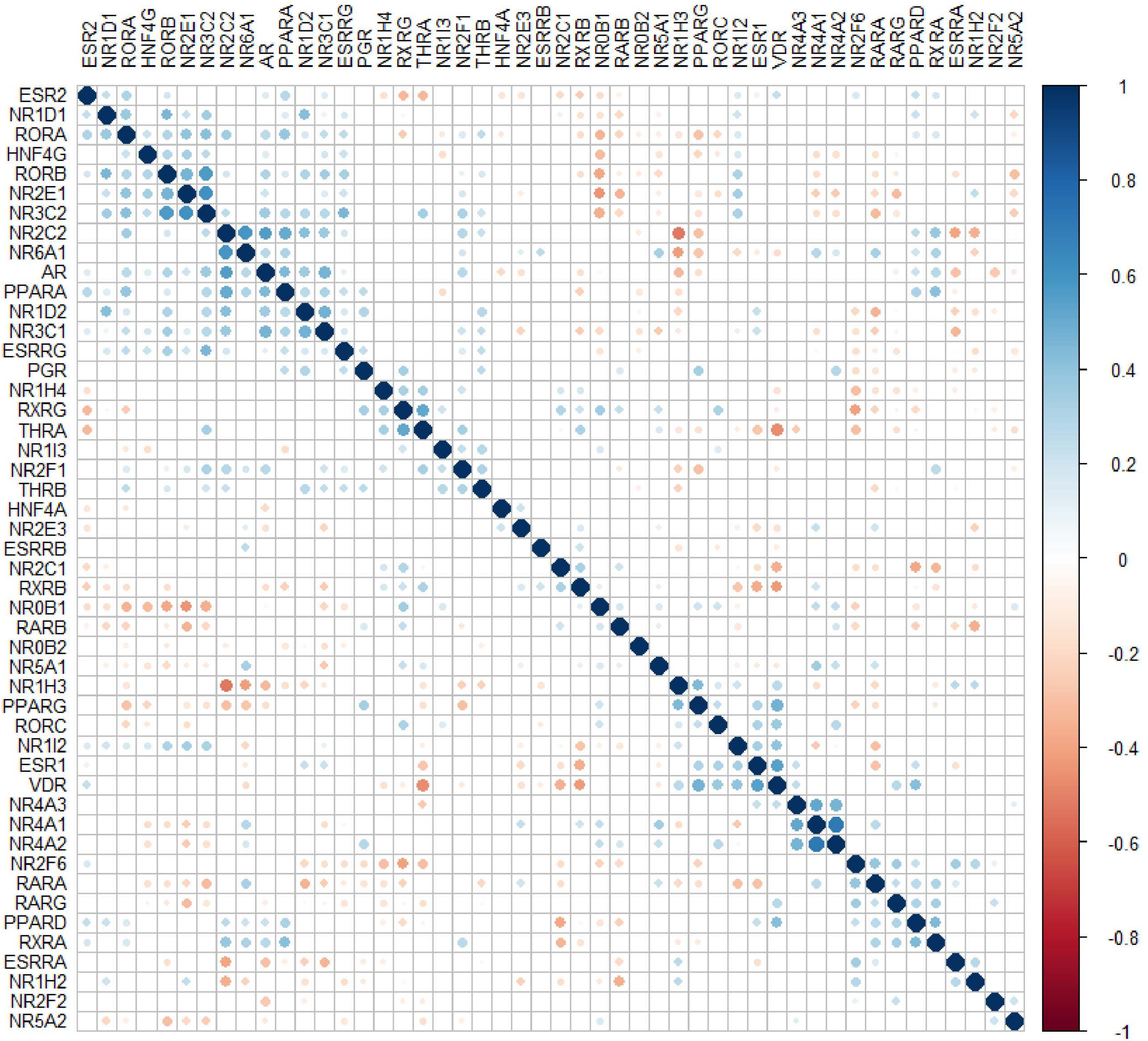

ESR2
NR1D1
RORA
HNF4G
RORB
NR2E1
NR3C2
NR2C2
NR6A1
AR
PPARA
NR1D2
NR3C1
ESRRG
PGR
NR1H4
RXRG
THRA
NR1I3
NR2F1
THRB
HNF4A
NR2E3
ESRRB
NR2C1
RXRB
NR0B1
RARB
NR0B2
NR5A1
NR1H3
PPARG
RORC
NR1I2
ESR1
VDR
NR4A3
NR4A1
NR4A2
NR2F6
RARA
RARG
PPARD
RXRA
ESRRA
NR1H2
NR2F2
NR5A2
1
0.8
0.6
0.4
0.2
0
-0.2
-0.4
-0.6
-0.8
-1

HNSC

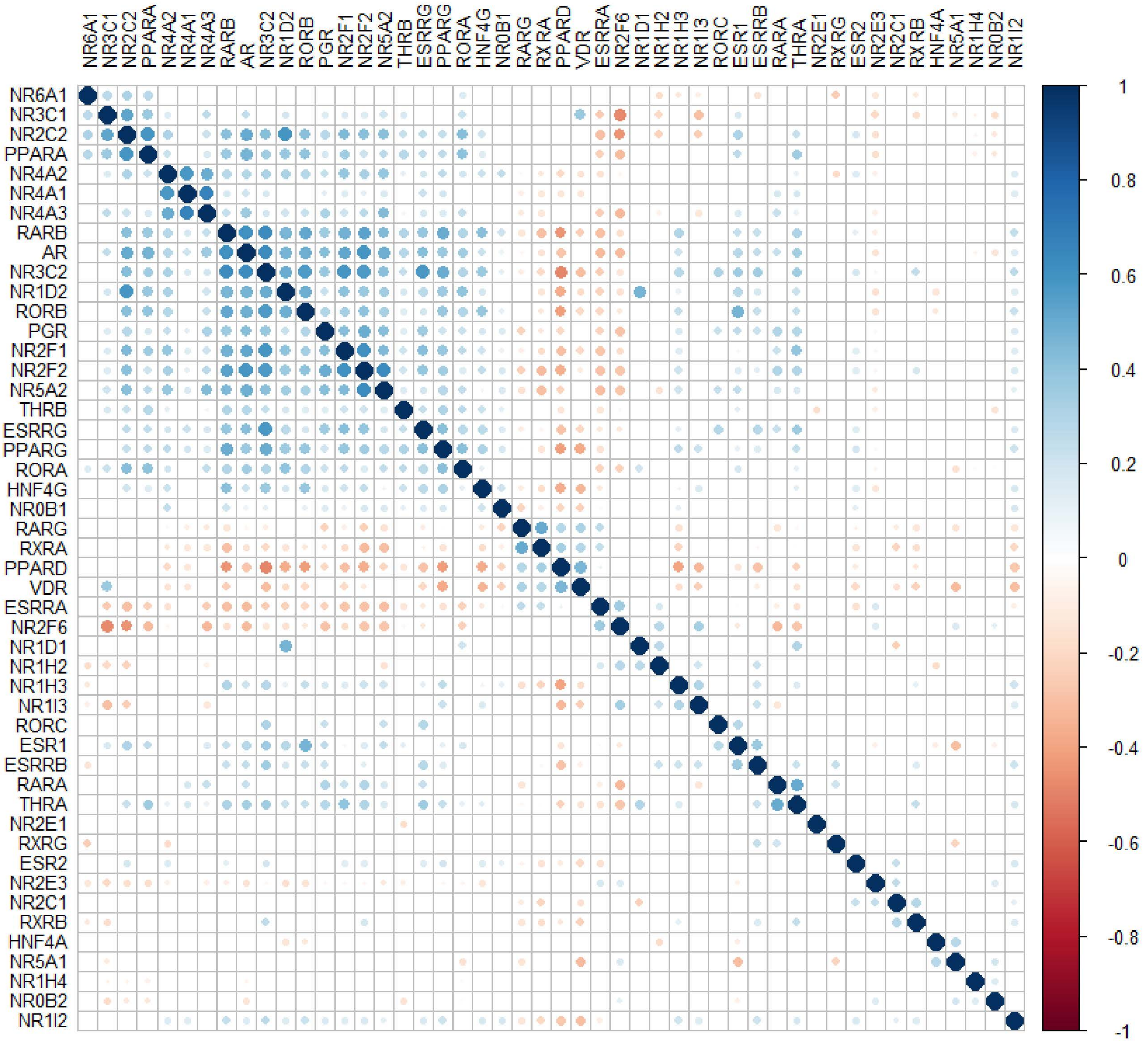
NR6A1
NR3C1
NR2C2
PPARA
NR4A2
NR4A1
NR4A3
RARB
AR
NR3C2
NR1D2
RORB
PGR
NR2F1
NR2F2
NR5A2
THRB
ESRRG
PPARG
RORA
HNF4G
NR0B1
RARG
RXRA
PPARD
VDR
ESRRA
NR2F6
NR1D1
NR1H2
NR1H3
NR1I3
RORC
ESR1
ESRRB
RARA
THRA
NR2E1
RXRG
ESR2
NR2E3
NR2C1
RXRB
HNF4A
NR5A1
NR1H4
NR0B2
NR1I2
1
0.8
0.6
0.4
0.2
0
-0.2
-0.4
-0.6
-0.8
-1

KIHC

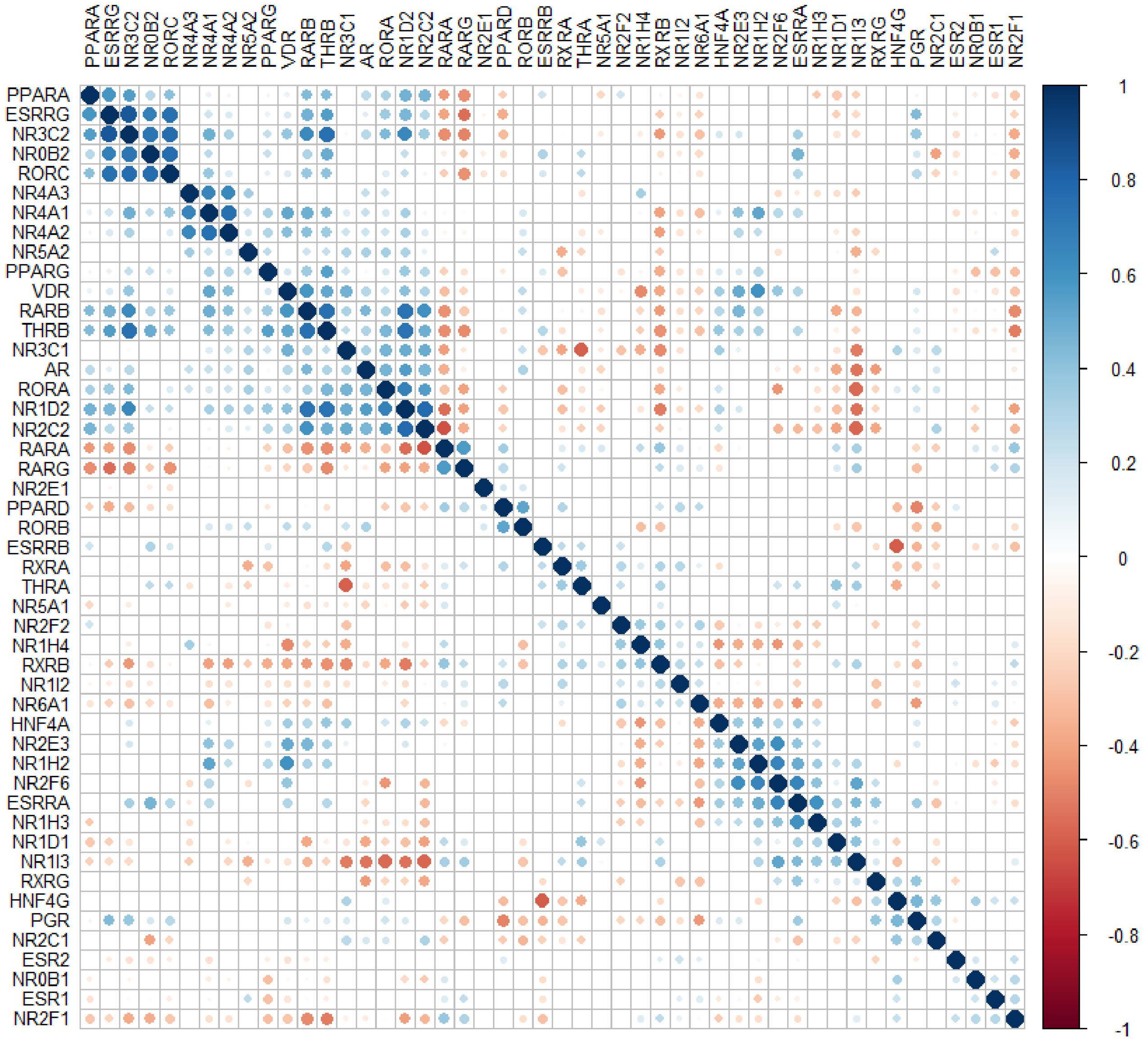

PPARA
ESRRG
NR3C2
NR0B2
RORC
NR4A3
NR4A1
NR4A2
NR5A2
PPARG
VDR
RARB
THRB
NR3C1
AR
RORA
NR1D2
NR2C2
RARA
RARG
NR2E1
PPARD
RORB
ESRRB
RXRA
THRA
NR5A1
NR2F2
NR1H4
RXRB
NR1I2
NR6A1
HNF4A
NR2E3
NR1H2
NR2F6
ESRRA
NR1H3
NR1D1
NR1I3
RXRG
HNF4G
PGR
NR2C1
ESR2
NR0B1
ESR1
NR2F1
1
0.8
0.6
0.4
0.2
0
-0.2
-0.4
-0.6
-0.8
-1

KIRC

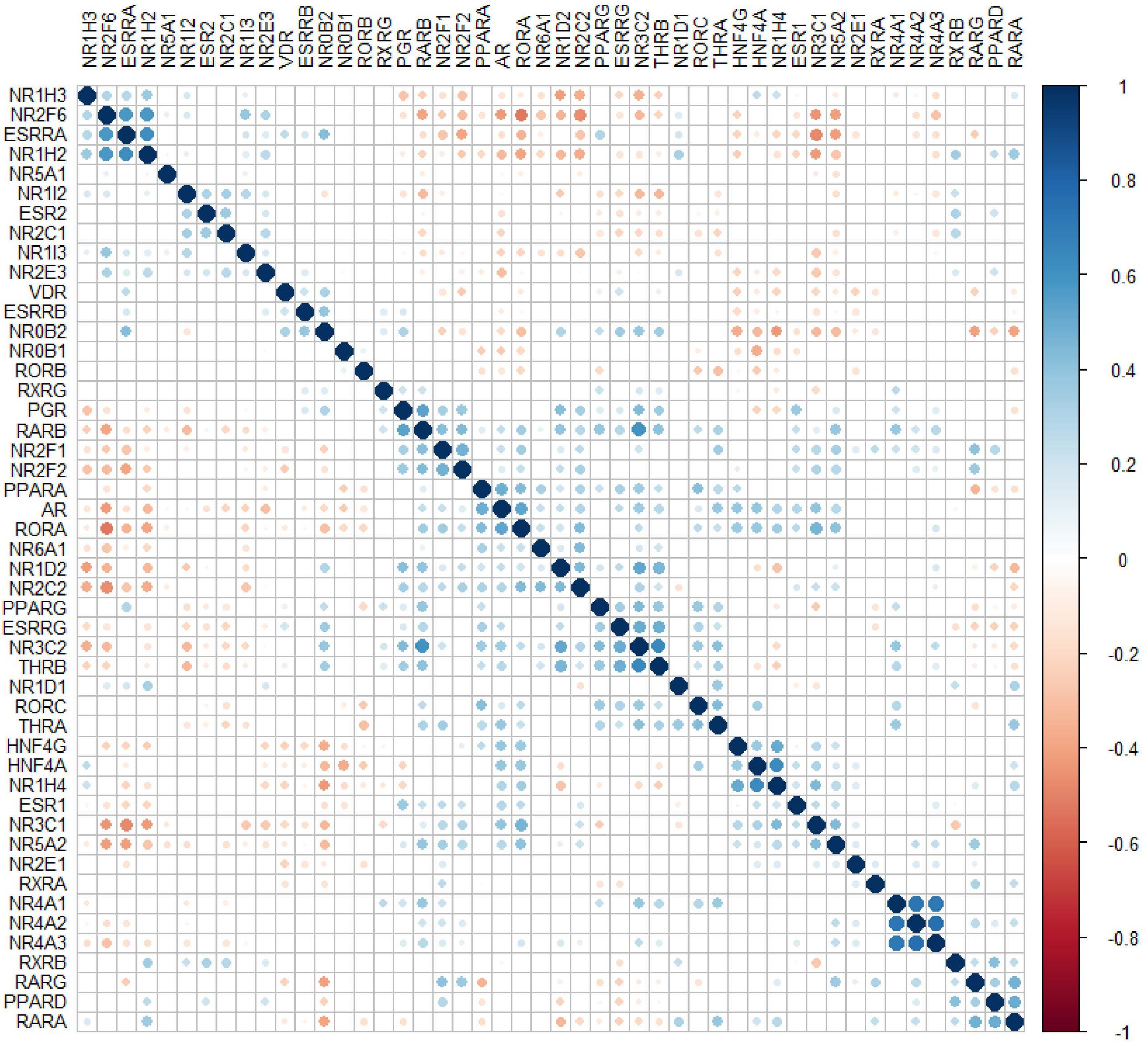

NR1H3
NR2F6
ESRRA
NR1H2
NR5A1
NR1I2
ESR2
NR2C1
NR1I3
NR2E3
VDR
ESRRB
NR0B2
NR0B1
RORB
RXRG
PGR
RARB
NR2F1
NR2F2
PPARA
AR
RORA
NR6A1
NR1D2
NR2C2
PPARG
ESRRG
NR3C2
THRB
NR1D1
RORC
THRA
HNF4G
HNF4A
NR1H4
ESR1
NR3C1
NR5A2
NR2E1
RXRA
NR4A1
NR4A2
NR4A3
RXRB
RARG
PPARD
RARA
1
0.8
0.6
0.4
0.2
0
-0.2
-0.4
-0.6
-0.8
-1

KIRP

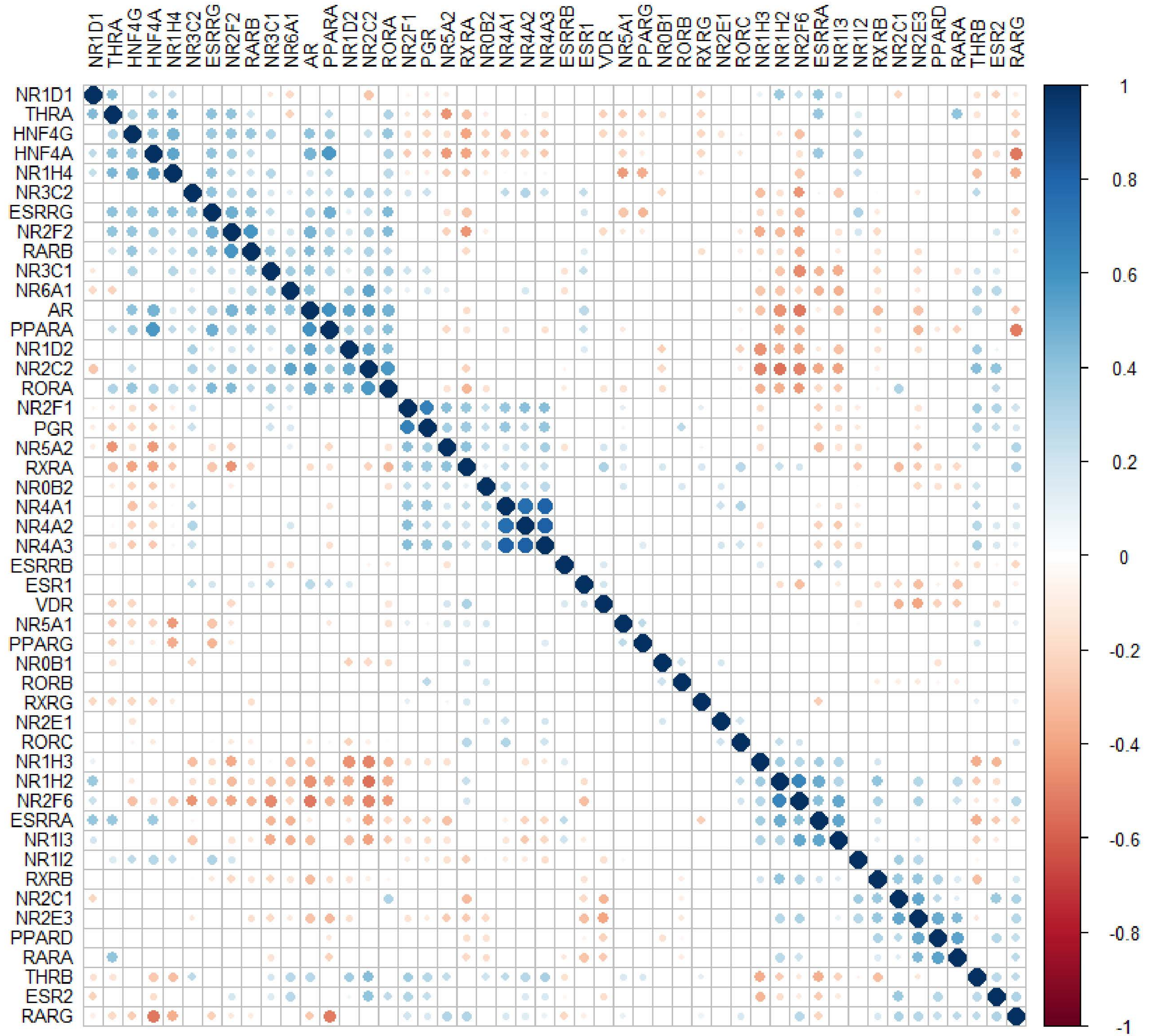
NR1D1
THRA
HNF4G
HNF4A
NR1H4
NR3C2
ESRRG
NR2F2
RARB
NR3C1
NR6A1
AR
PPARA
NR1D2
NR2C2
RORA
NR2F1
PGR
NR5A2
RXRA
NR0B2
NR4A1
NR4A2
NR4A3
ESRRB
ESR1
VDR
NR5A1
PPARG
NR0B1
RORB
RXRG
NR2E1
RORC
NR1H3
NR1H2
NR2F6
ESRRA
NR1I3
NR1I2
RXRB
NR2C1
NR2E3
PPARD
RARA
THRB
ESR2
RARG
1
0.8
0.6
0.4
0.2
0
-0.2
-0.4
-0.6
-0.8
-1

LIHC

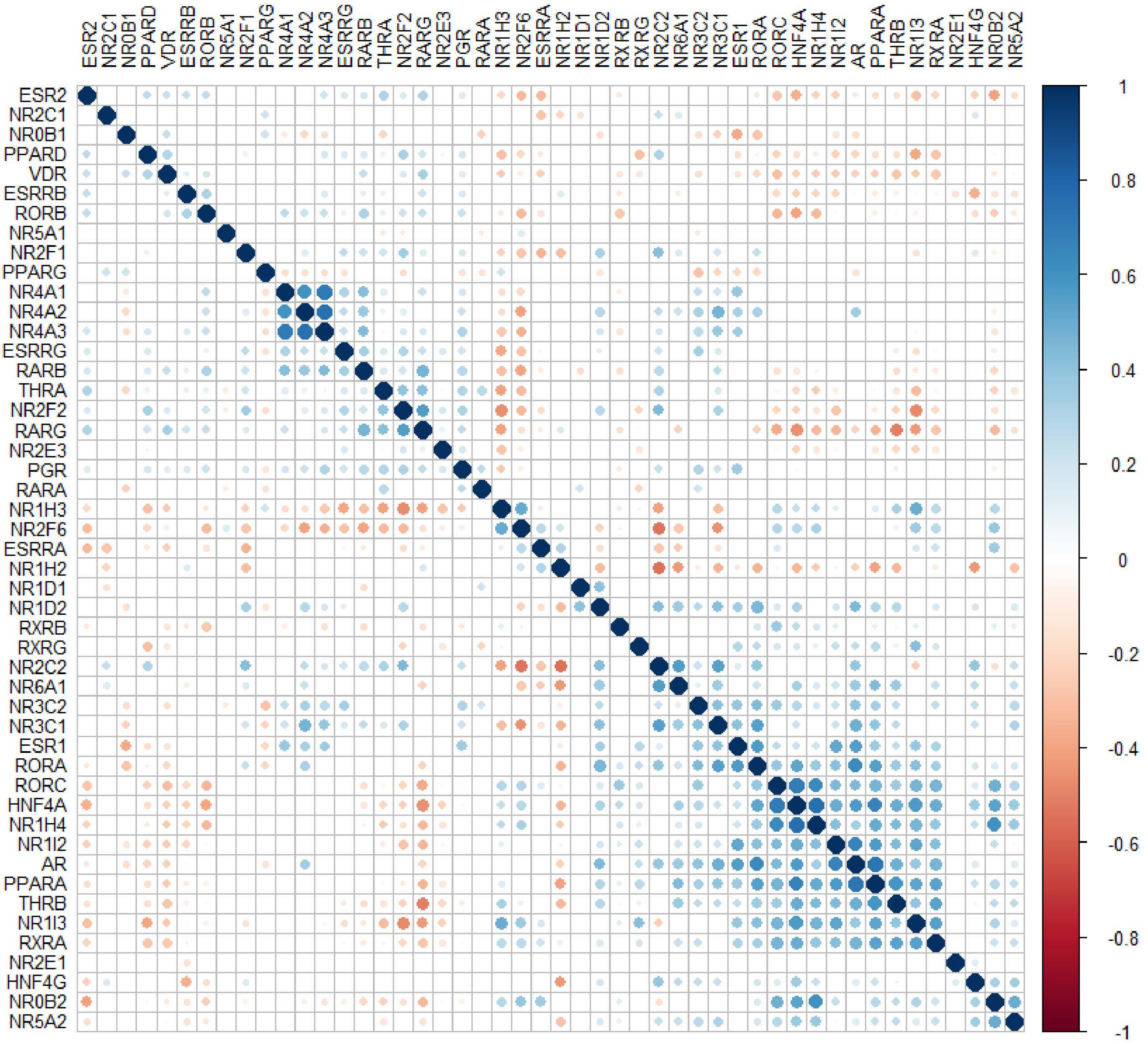
ESR2
NR2C1
NR0B1
PPARD
VDR
ESRRB
RORB
NR5A1
NR2F1
PPARG
NR4A1
NR4A2
NR4A3
ESRRG
RARB
THRA
NR2F2
RARG
NR2E3
PGR
RARA
NR1H3
NR2F6
ESRRA
NR1H2
NR1D1
NR1D2
RXRB
RXRG
NR2C2
NR6A1
NR3C2
NR3C1
ESR1
RORA
RORC
HNF4A
NR1H4
NR1I2
AR
PPARA
THRB
NR1I3
RXRA
NR2E1
HNF4G
NR0B2
NR5A2
1
0.8
0.6
0.4
0.2
0
-0.2
-0.4
-0.6
-0.8
-1

LUAD

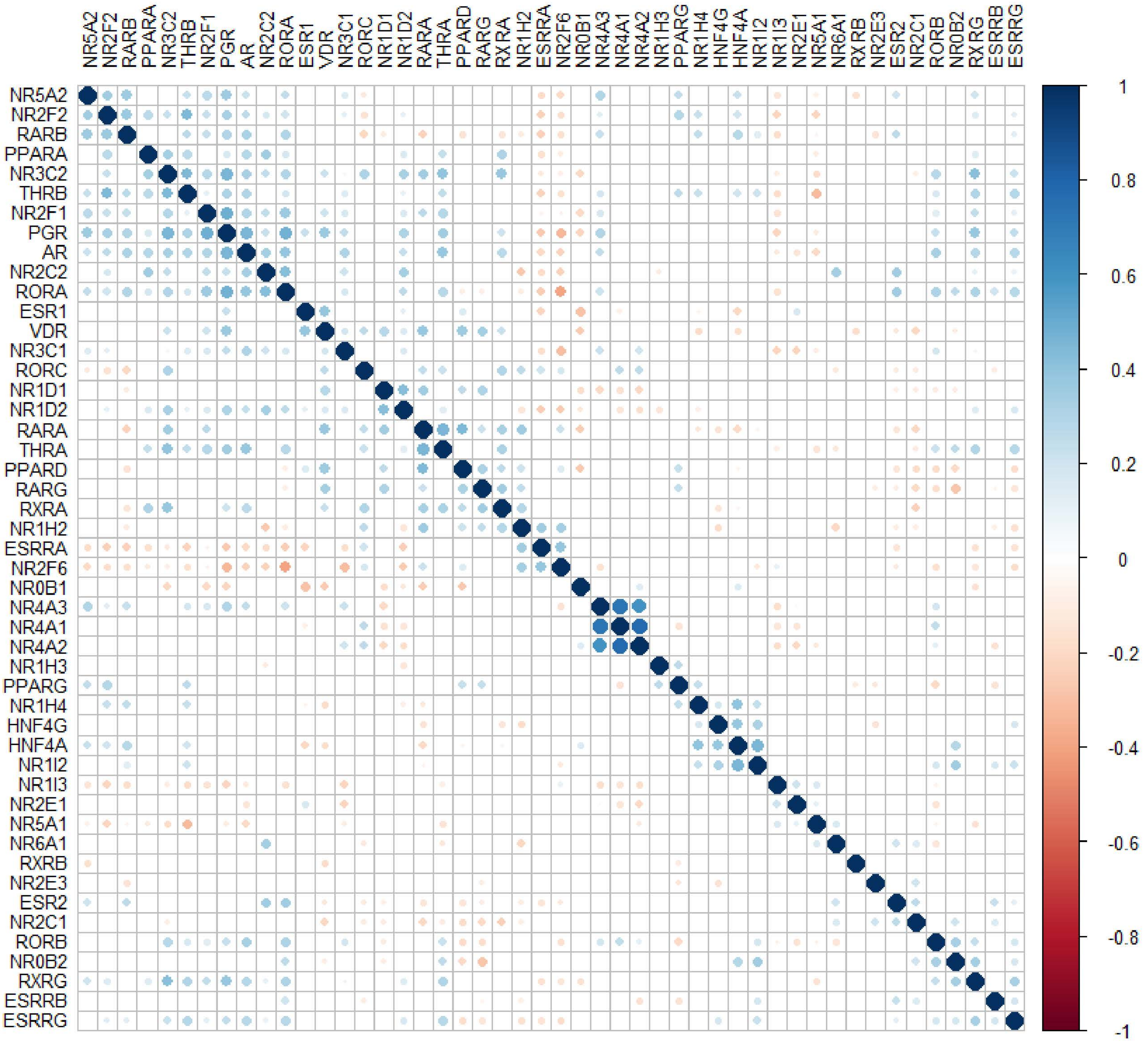
NR5A2
NR2F2
RARB
PPARA
NR3C2
THRB
NR2F1
PGR
AR
NR2C2
RORA
ESR1
VDR
NR3C1
RORC
NR1D1
NR1D2
RARA
THRA
PPARD
RARG
RXRA
NR1H2
ESRRA
NR2F6
NR0B1
NR4A3
NR4A1
NR4A2
NR1H3
PPARG
NR1H4
HNF4G
HNF4A
NR1I2
NR1I3
NR2E1
NR5A1
NR6A1
RXRB
NR2E3
ESR2
NR2C1
RORB
NR0B2
RXRG
ESRRB
ESRRG
1
0.8
0.6
0.4
0.2
0
-0.2
-0.4
-0.6
-0.8
-1

LUSC

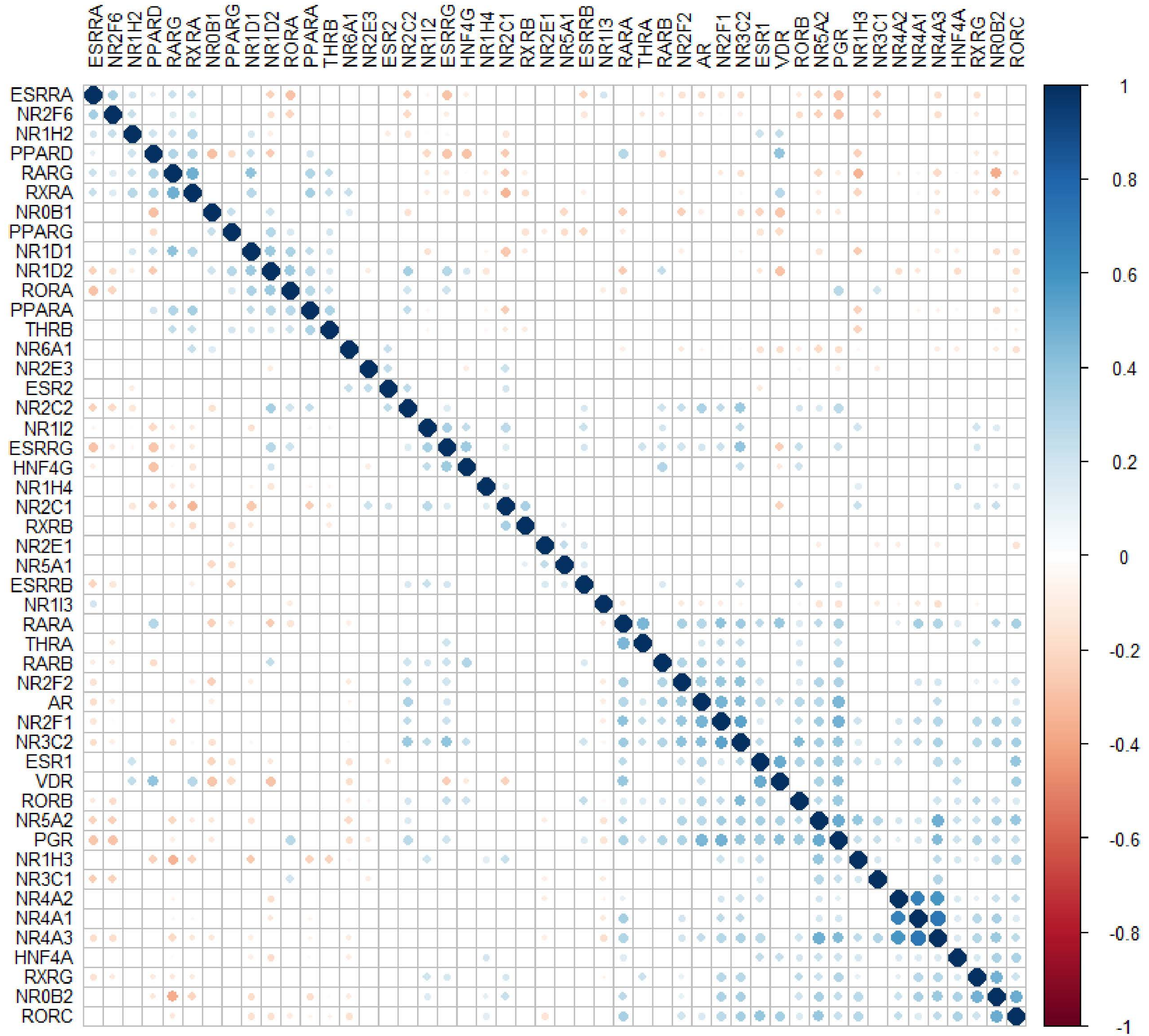
ESRRA
NR2F6
NR1H2
PPARD
RARG
RXRA
NR0B1
PPARG
NR1D1
NR1D2
RORA
PPARA
THRB
NR6A1
NR2E3
ESR2
NR2C2
NR1I2
ESRRG
HNF4G
NR1H4
NR2C1
RXRB
NR2E1
NR5A1
ESRRB
NR1I3
RARA
THRA
RARB
NR2F2
AR
NR2F1
NR3C2
ESR1
VDR
RORB
NR5A2
PGR
NR1H3
NR3C1
NR4A2
NR4A1
NR4A3
HNF4A
RXRG
NR0B2
RORC
1
0.8
0.6
0.4
0.2
0
-0.2
-0.4
-0.6
-0.8
-1

PAAD

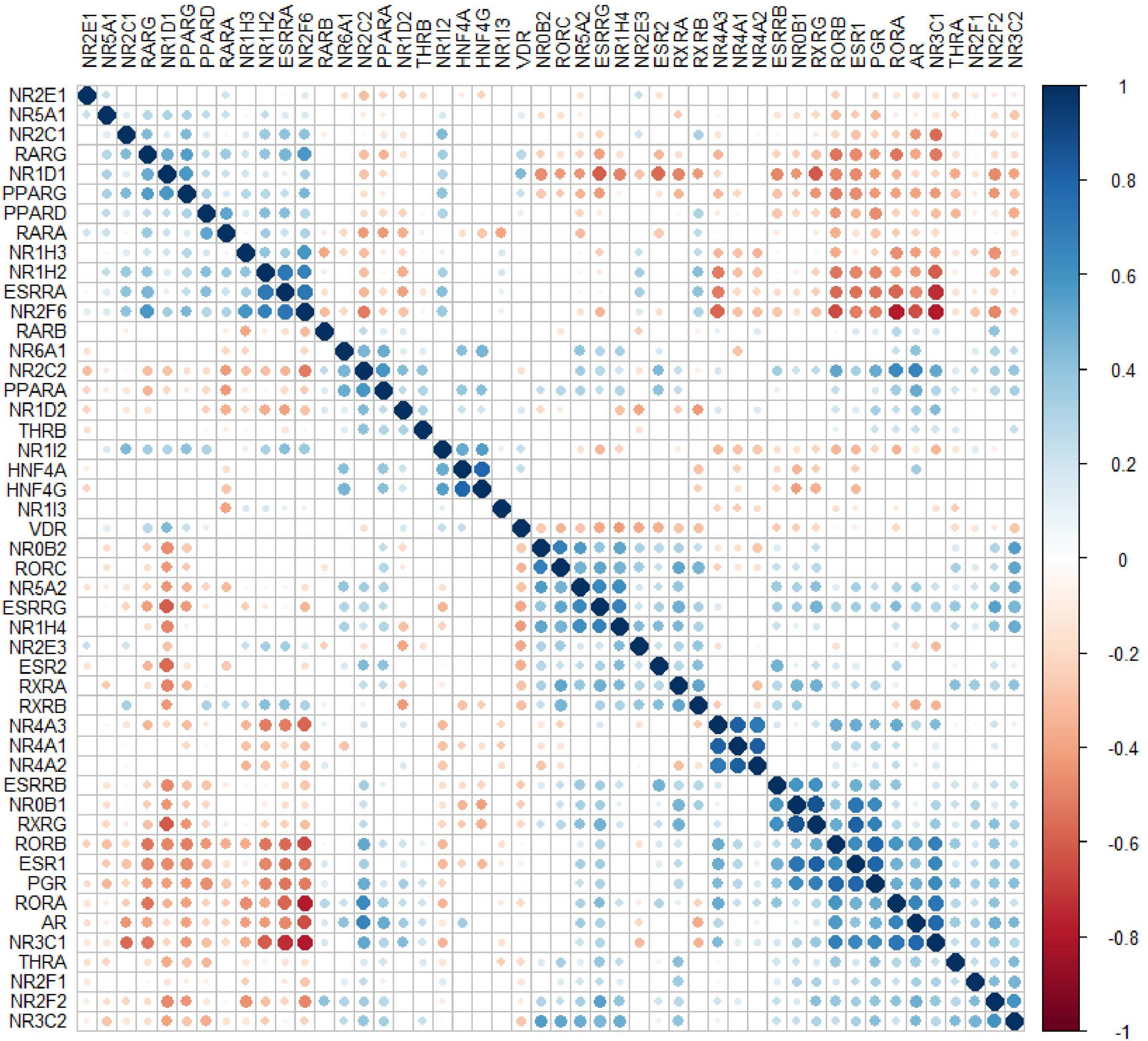

NR2E1
NR5A1
NR2C1
RARG
NR1D1
PPARG
PPARD
RARA
NR1H3
NR1H2
ESRRA
NR2F6
RARB
NR6A1
NR2C2
PPARA
NR1D2
THRB
NR1I2
HNF4A
HNF4G
NR1I3
VDR
NR0B2
RORC
NR5A2
ESRRG
NR1H4
NR2E3
ESR2
RXRA
RXRB
NR4A3
NR4A1
NR4A2
ESRRB
NR0B1
RXRG
RORB
ESR1
PGR
RORA
AR
NR3C1
THRA
NR2F1
NR2F2
NR3C2
1
0.8
0.6
0.4
0.2
0
-0.2
-0.4
-0.6
-0.8
-1

PCPG

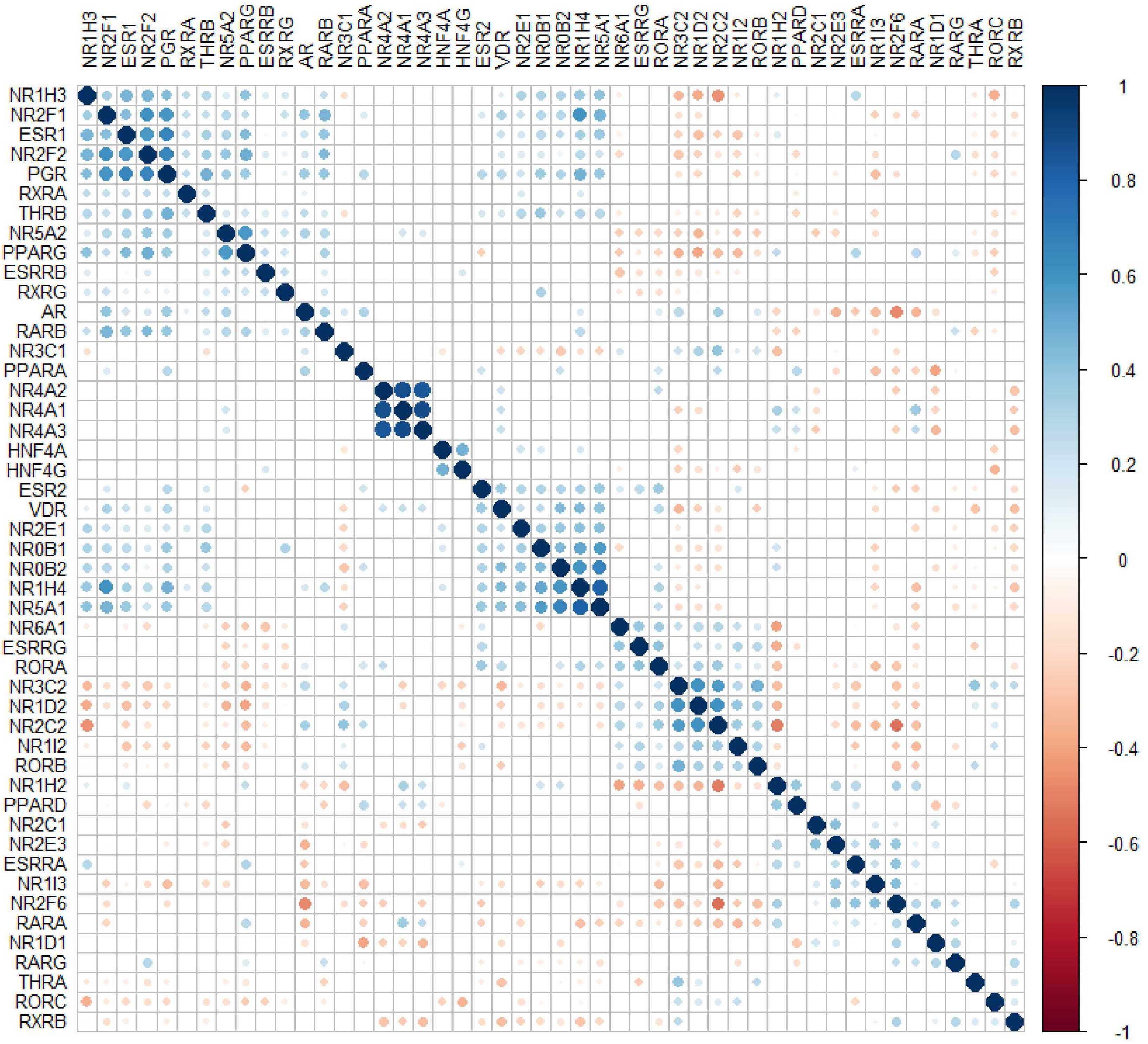

NR1H3
NR2F1
ESR1
NR2F2
PGR
RXRA
THRB
NR5A2
PPARG
ESRRB
RXRG
AR
RARB
NR3C1
PPARA
NR4A2
NR4A1
NR4A3
HNF4A
HNF4G
ESR2
VDR
NR2E1
NR0B1
NR0B2
NR1H4
NR5A1
NR6A1
ESRRG
RORA
NR3C2
NR1D2
NR2C2
NR1I2
RORB
NR1H2
PPARD
NR2C1
NR2E3
ESRRA
NR1I3
NR2F6
RARA
NR1D1
RARG
THRA
RORC
RXRB
1
0.8
0.6
0.4
0.2
0
-0.2
-0.4
-0.6
-0.8
-1

PRAD

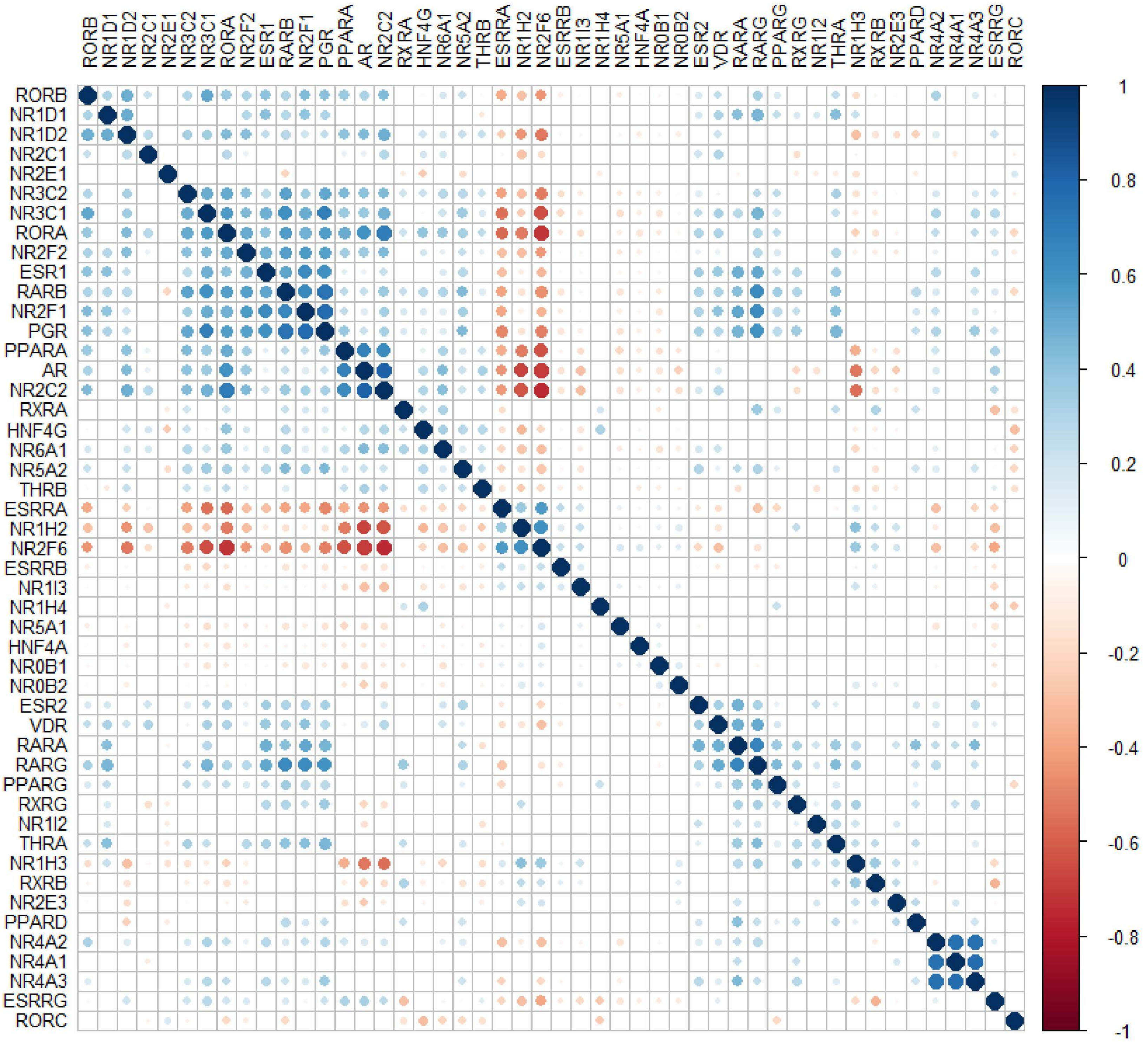
RORB
NR1D1
NR1D2
NR2C1
NR2E1
NR3C2
NR3C1
RORA
NR2F2
ESR1
RARB
NR2F1
PGR
PPARA
AR
NR2C2
RXRA
HNF4G
NR6A1
NR5A2
THRB
ESRRA
NR1H2
NR2F6
ESRRB
NR1I3
NR1H4
NR5A1
HNF4A
NR0B1
NR0B2
ESR2
VDR
RARA
RARG
PPARG
RXRG
NR1I2
THRA
NR1H3
RXRB
NR2E3
PPARD
NR4A2
NR4A1
NR4A3
ESRRG
RORC
1
0.8
0.6
0.4
0.2
0
-0.2
-0.4
-0.6
-0.8
-1

SARC

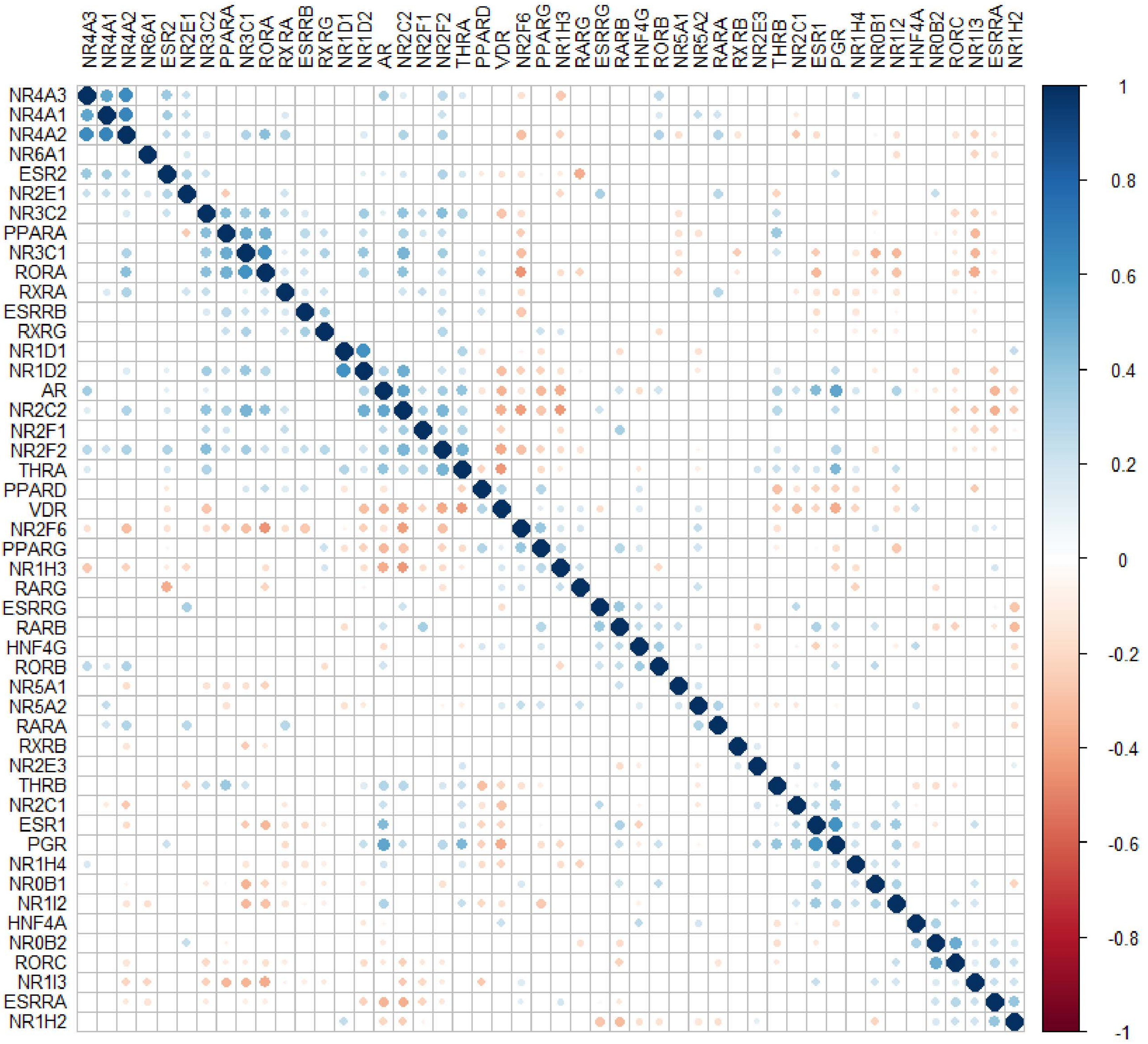
NR4A3
NR4A1
NR4A2
NR6A1
ESR2
NR2E1
NR3C2
PPARA
NR3C1
RORA
RXRA
ESRRB
RXRG
NR1D1
NR1D2
AR
NR2C2
NR2F1
NR2F2
THRA
PPARD
VDR
NR2F6
PPARG
NR1H3
RARG
ESRRG
RARB
HNF4G
RORB
NR5A1
NR5A2
RARA
RXRB
NR2E3
THRB
NR2C1
ESR1
PGR
NR1H4
NR0B1
NR1I2
HNF4A
NR0B2
RORC
NR1I3
ESRRA
NR1H2
1
0.8
0.6
0.4
0.2
0
-0.2
-0.4
-0.6
-0.8
-1

SKCM

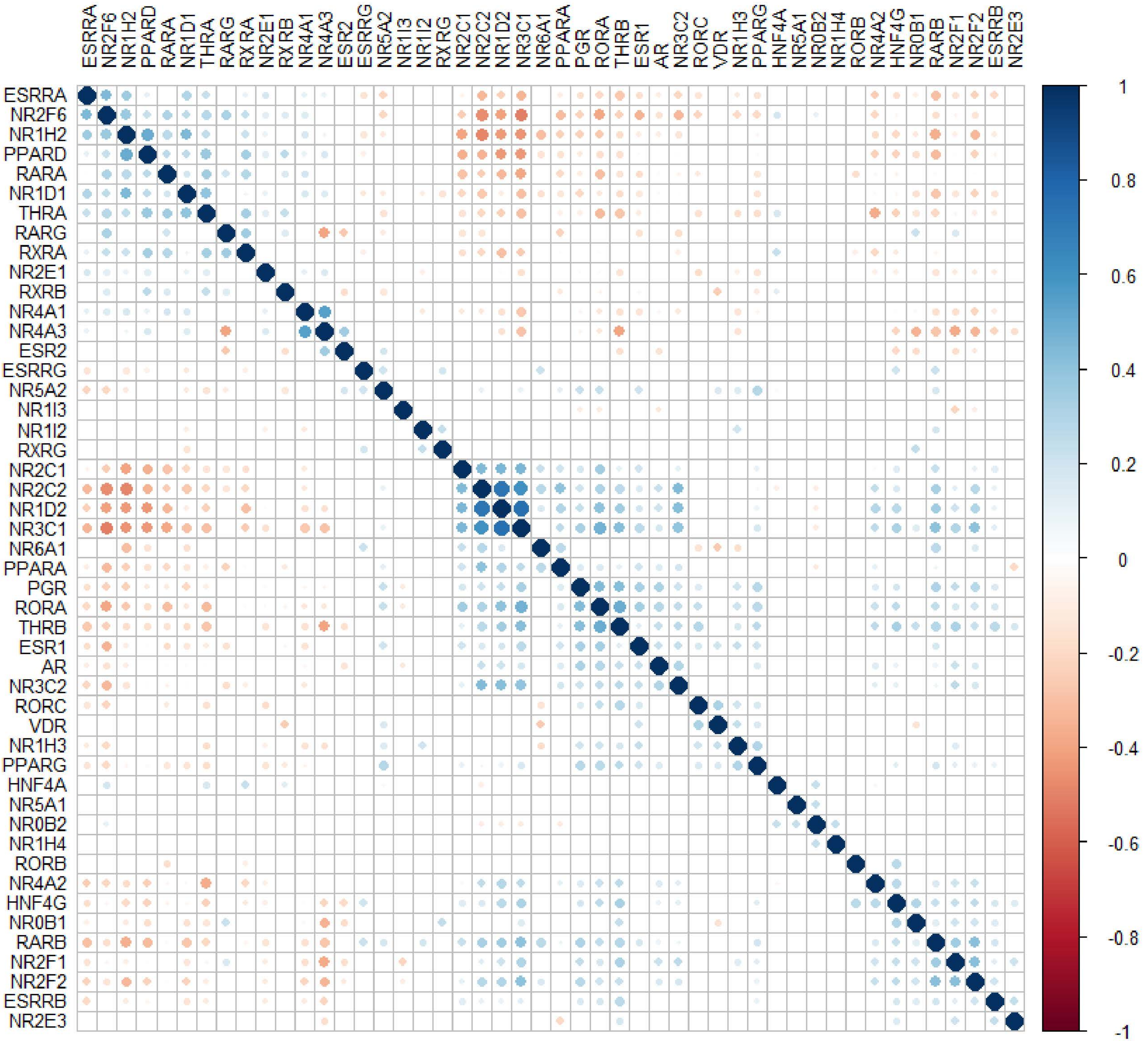
ESRRA
NR2F6
NR1H2
PPARD
RARA
NR1D1
THRA
RARG
RXRA
NR2E1
RXRB
NR4A1
NR4A3
ESR2
ESRRG
NR5A2
NR1I3
NR1I2
RXRG
NR2C1
NR2C2
NR1D2
NR3C1
NR6A1
PPARA
PGR
RORA
THRB
ESR1
AR
NR3C2
RORC
VDR
NR1H3
PPARG
HNF4A
NR5A1
NR0B2
NR1H4
RORB
NR4A2
HNF4G
NR0B1
RARB
NR2F1
NR2F2
ESRRB
NR2E3
1
0.8
0.6
0.4
0.2
0
-0.2
-0.4
-0.6
-0.8
-1

STAD

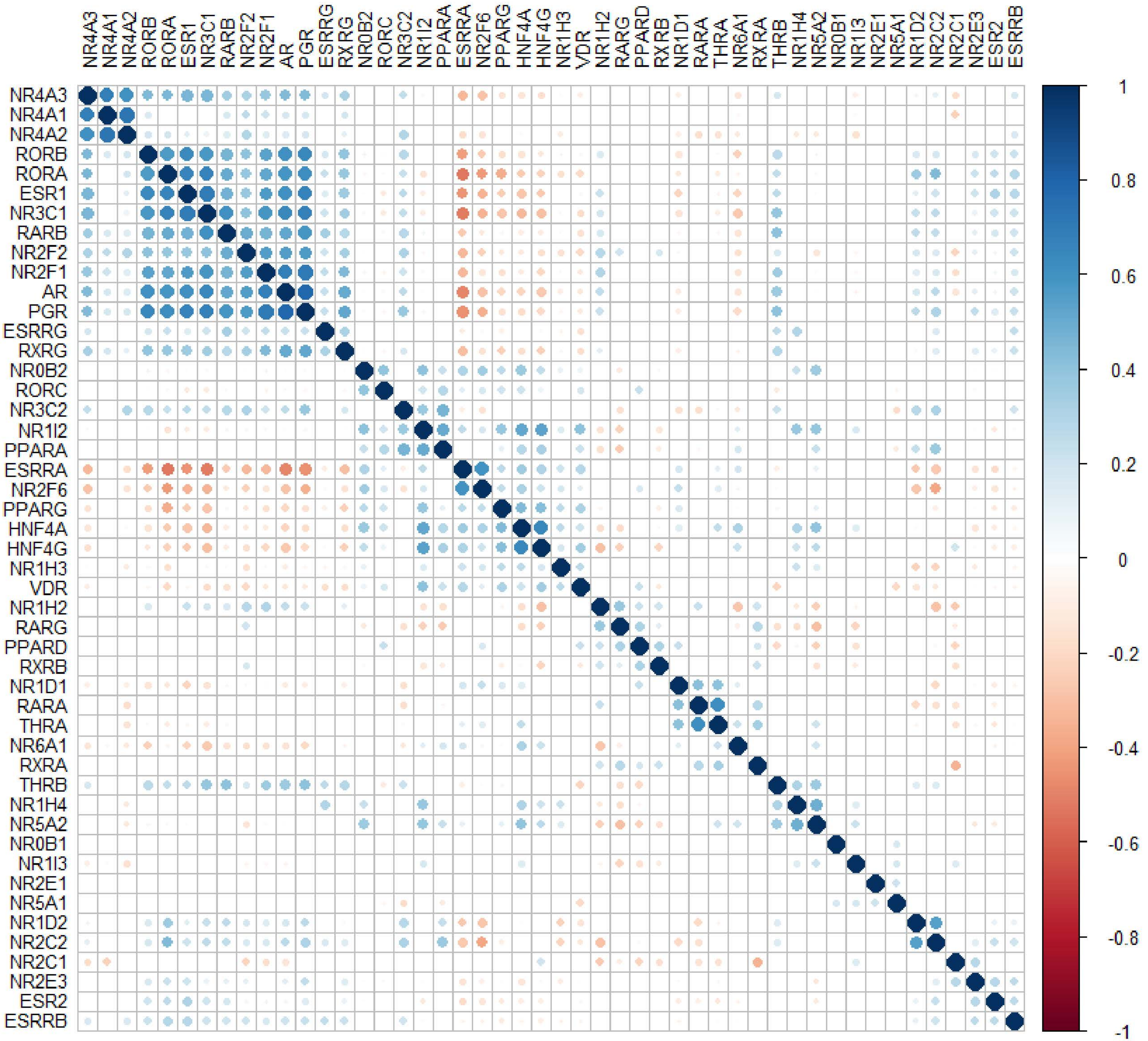

NR4A3
NR4A1
NR4A2
RORB
RORA
ESR1
NR3C1
RARB
NR2F2
NR2F1
AR
PGR
ESRRG
RXRG
NR0B2
RORC
NR3C2
NR1I2
PPARA
ESRRA
NR2F6
PPARG
HNF4A
HNF4G
NR1H3
VDR
NR1H2
RARG
PPARD
RXRB
NR1D1
RARA
THRA
NR6A1
RXRA
THRB
NR1H4
NR5A2
NR0B1
NR1I3
NR2E1
NR5A1
NR1D2
NR2C2
NR2C1
NR2E3
ESR2
ESRRB
1
0.8
0.6
0.4
0.2
0
-0.2
-0.4
-0.6
-0.8
-1

THCA

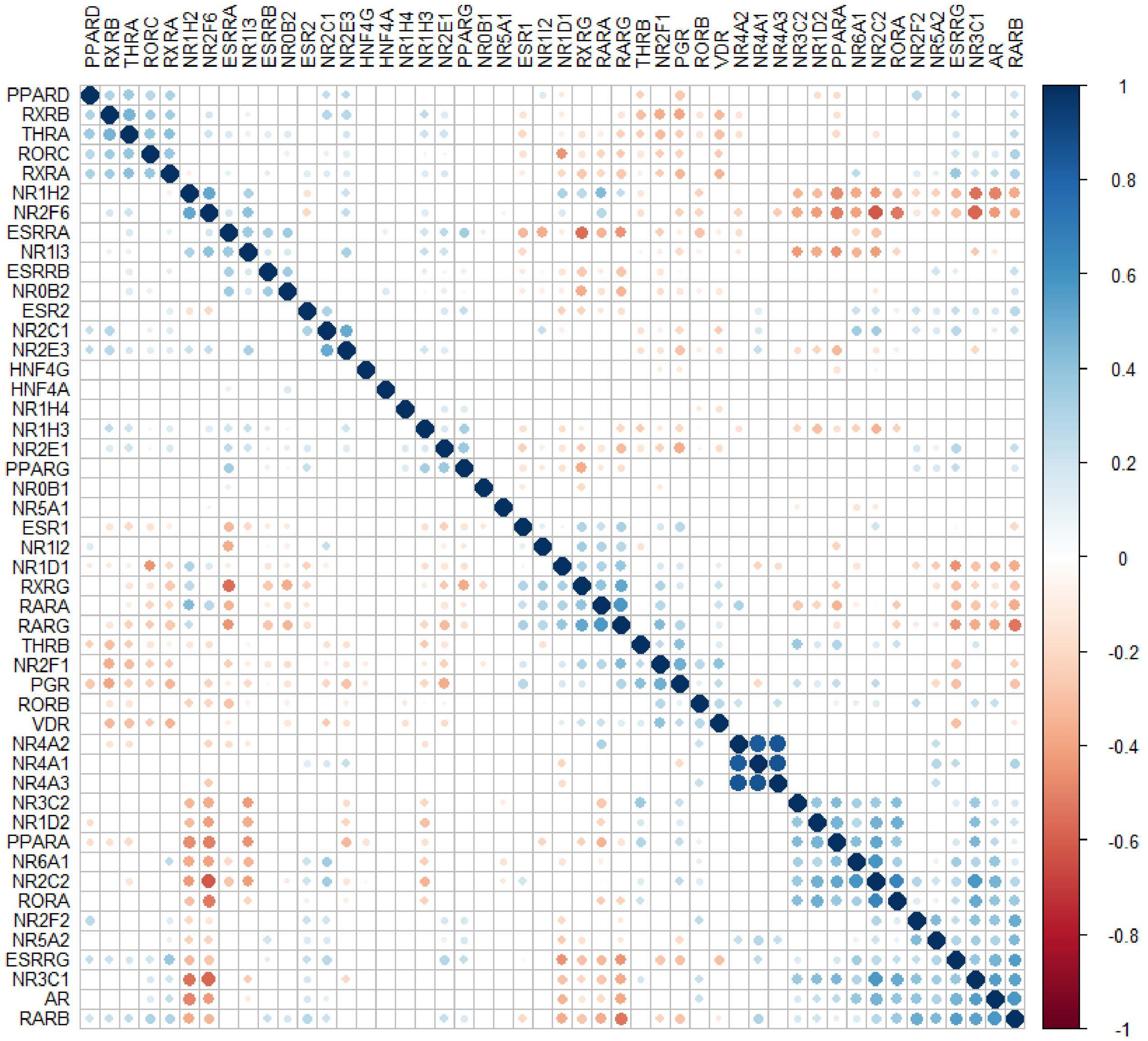

PPARD
RXRB
THRA
RORC
RXRA
NR1H2
NR2F6
ESRRA
NR1I3
ESRRB
NR0B2
ESR2
NR2C1
NR2E3
HNF4G
HNF4A
NR1H4
NR1H3
NR2E1
PPARG
NR0B1
NR5A1
ESR1
NR1I2
NR1D1
RXRG
RARA
RARG
THRB
NR2F1
PGR
RORB
VDR
NR4A2
NR4A1
NR4A3
NR3C2
NR1D2
PPARA
NR6A1
NR2C2
RORA
NR2F2
NR5A2
ESRRG
NR3C1
AR
RARB
1
0.8
0.6
0.4
0.2
0
-0.2
-0.4
-0.6
-0.8
-1

THYM

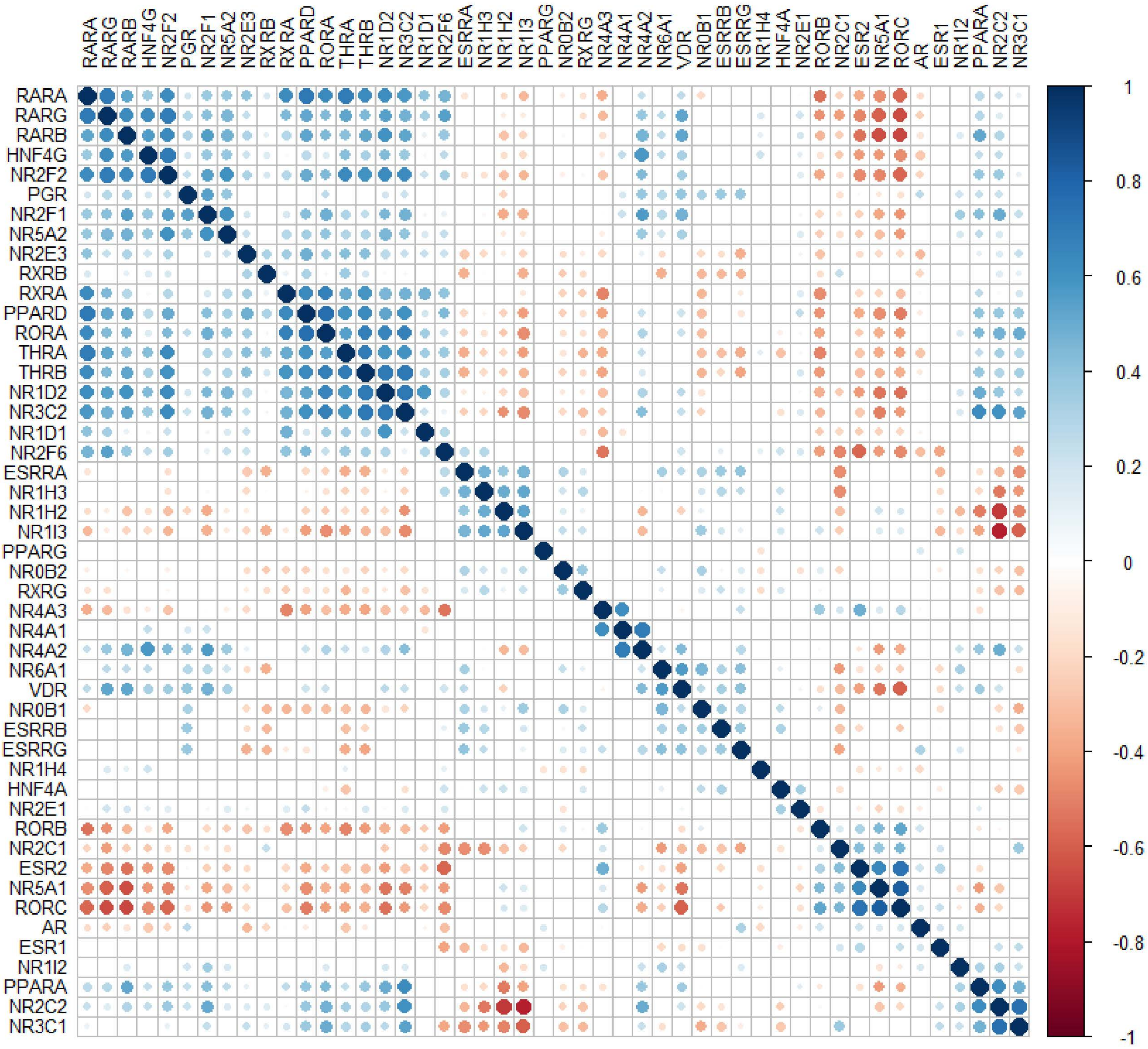

RARA
RARG
RARB
HNF4G
NR2F2
PGR
NR2F1
NR5A2
NR2E3
RXRB
RXRA
PPARD
RORA
THRA
THRB
NR1D2
NR3C2
NR1D1
NR2F6
ESRRA
NR1H3
NR1H2
NR1I3
PPARG
NR0B2
RXRG
NR4A3
NR4A1
NR4A2
NR6A1
VDR
NR0B1
ESRRB
ESRRG
NR1H4
HNF4A
NR2E1
RORB
NR2C1
ESR2
NR5A1
RORC
AR
ESR1
NR1I2
PPARA
NR2C2
NR3C1
1
0.8
0.6
0.4
0.2
0
-0.2
-0.4
-0.6
-0.8
-1

Supplementary Table 1. NR gene expression levels in TCGA cancer types and corresponding pan-cancer organ systems

		Class I																		
**Disease name and pan-organ system**	**Cohort**	***THRA***	***THRB***	***RARA***	***RARB***	***RARG***	***PPARA***	***PPARD***	***PPARG***	***NR1D1***	***NR1D2***	***RORA***	***RORB***	***RORC***	***NR1H2***	***NR1H3***	***NR1H4***	***VDR***	***NR1I2***	***NR1I3***
Central nervous system (CNS)																				
Glioblastoma multiforme	GBM	high	moderate	high	low	moderate	moderate	high	low	moderate	moderate	moderate	low	absent	high	moderate	absent	low	absent	low
Brain lower grade glioma	LGG	high	moderate	moderate	low	low	moderate	high	low	moderate	high	moderate	low	absent	high	moderate	absent	absent	absent	low
Endocrine																				
Adrenocortical carcinoma	ACC	high	low	moderate	low	moderate	moderate	high	low	high	high	high	low	low	high	low	moderate	high	absent	absent
Thyroid carcinoma	THCA	high	low	high	low	high	moderate	high	low	high	high	moderate	absent	high	high	moderate	absent	moderate	absent	absent
Gastrointestinal																				
Cholangiocarcinoma	CHOL	moderate	low	high	low	moderate	moderate	high	low	moderate	moderate	moderate	absent	moderate	high	moderate	moderate	low	low	low
Colon adenocarcinoma	COAD	high	low	moderate	low	moderate	high	high	high	moderate	moderate	low	absent	moderate	high	moderate	absent	high	moderate	absent
Esophageal carcinoma	ESCA	moderate	moderate	high	low	high	high	high	low	high	high	moderate	absent	low	high	moderate	absent	high	low	absent
Liver hepatocellular carcinoma	LIHC	moderate	moderate	high	low	low	high	high	moderate	high	high	moderate	absent	high	high	high	high	low	moderate	moderate
Pancreatic adenocarcinoma	PAAD	high	moderate	high	moderate	high	moderate	high	moderate	moderate	high	moderate	low	moderate	high	moderate	low	high	low	absent
Rectum adenocarcinoma	READ	high	low	high	low	moderate	high	high	high	moderate	moderate	low	absent	moderate	high	moderate	absent	high	moderate	absent
Stomach adenocarcinoma	STAD	high	moderate	high	low	moderate	high	high	moderate	moderate	high	moderate	absent	low	high	moderate	absent	high	low	absent
Gynecologic																				
Breast invasive carcinoma	BRCA	moderate	moderate	high	low	high	moderate	high	low	moderate	high	moderate	absent	moderate	high	moderate	absent	high	absent	absent
Cervical and endocervical cancers	CESC	moderate	low	moderate	low	high	moderate	high	low	moderate	moderate	moderate	absent	low	high	moderate	absent	high	absent	low
Ovarian serous cystadenocarcinoma	OV	high	moderate	high	low	high	moderate	high	low	moderate	high	low	absent	moderate	high	moderate	absent	moderate	low	absent
Uterine corpus endometrial carcinoma	UCEC	moderate	low	high	low	moderate	moderate	high	low	moderate	moderate	low	absent	moderate	high	moderate	absent	moderate	absent	absent
Head and neck																				
Head and Neck squamous cell carcinoma	HNSC	moderate	low	moderate	low	high	moderate	high	low	moderate	moderate	moderate	absent	low	high	moderate	absent	high	absent	absent
Hematologic and lymphatic malignancies																				
Lymphoid neoplasm diffuse large B-cell lymphoma	DLBC	low	low	moderate	low	low	moderate	moderate	low	moderate	moderate	low	absent	absent	high	high	absent	moderate	low	low
Acute myeloid leukemia	LAML	moderate	low	high	low	low	high	moderate	low	low	moderate	moderate	absent	absent	high	moderate	absent	moderate	low	low
Thymoma	THYM	high	low	moderate	low	moderate	moderate	high	low	moderate	moderate	moderate	absent	moderate	high	moderate	absent	low	absent	absent
Melanocytic																				
Skin cutaneous melanoma	SKCM	moderate	low	high	low	high	moderate	high	low	moderate	moderate	low	absent	low	high	moderate	absent	moderate	absent	low
Uveal melanoma	UVM	high	absent	high	low	moderate	moderate	high	low	high	moderate	low	absent	low	high	moderate	absent	low	absent	absent
Neural crest-derived																				
Pheochromocytoma and Paraganglioma	PCPG	high	low	high	low	moderate	moderate	high	low	high	high	moderate	low	high	high	low	absent	low	absent	low
Soft tissue																				
Sarcoma	SARC	high	moderate	high	moderate	moderate	moderate	high	moderate	moderate	high	moderate	absent	absent	high	moderate	absent	low	absent	absent
Uterine carcinosarcoma	UCS	high	low	high	low	high	moderate	high	low	moderate	moderate	low	absent	low	high	moderate	absent	low	absent	absent
Thoracic																				
Lung adenocarcinoma	LUAD	moderate	low	high	low	moderate	moderate	high	moderate	moderate	high	moderate	absent	moderate	high	moderate	absent	high	absent	absent
Lung squamous cell carcinoma	LUSC	moderate	low	moderate	moderate	high	moderate	high	low	moderate	high	moderate	absent	low	high	moderate	absent	moderate	absent	absent
Mesothelioma	MESO	high	low	high	low	moderate	moderate	high	low	moderate	moderate	moderate	absent	low	high	moderate	absent	moderate	absent	absent
Urologic																				
Bladder urothelial carcinoma	BLCA	moderate	moderate	moderate	low	high	moderate	high	high	moderate	high	low	absent	low	high	moderate	absent	moderate	absent	low
Kidney chromophobe	KICH	high	high	moderate	moderate	low	high	high	moderate	high	high	moderate	absent	high	high	moderate	absent	high	absent	low
Kidney renal clear cell carcinoma	KIRC	high	moderate	high	moderate	moderate	high	high	moderate	moderate	moderate	high	absent	high	high	moderate	moderate	moderate	absent	absent
Kidney renal papillary cell carcinoma	KIRP	high	low	high	moderate	moderate	high	high	moderate	high	moderate	moderate	absent	moderate	high	moderate	moderate	moderate	absent	low
Prostate adenocarcinoma	PRAD	high	high	moderate	low	moderate	moderate	high	low	moderate	high	moderate	low	high	high	moderate	absent	low	absent	absent
Testicular Germ Cell Tumors	TGCT	moderate	low	moderate	moderate	moderate	low	high	moderate	moderate	moderate	moderate	absent	low	high	moderate	absent	low	absent	low

		Class II											
**Disease name and pan-organ system**	**Cohort**	***HNF4A***	***HNF4G***	***RXRA***	***RXRB***	***RXRG***	***NR2C1***	***NR2C2***	***NR2E1***	***NR2E3***	***NR2F1***	***NR2F2***	***NR2F6***
Central nervous system (CNS)													
Glioblastoma multiforme	GBM	absent	low	high	high	low	moderate	high	moderate	absent	high	moderate	moderate
Brain lower grade glioma	LGG	absent	low	high	high	low	moderate	high	low	absent	high	moderate	moderate
Endocrine													
Adrenocortical carcinoma	ACC	absent	absent	high	high	absent	moderate	moderate	absent	absent	low	high	high
Thyroid carcinoma	THCA	absent	absent	high	high	high	moderate	high	absent	absent	low	moderate	high
Gastrointestinal													
Cholangiocarcinoma	CHOL	moderate	moderate	high	high	absent	moderate	moderate	absent	absent	low	high	high
Colon adenocarcinoma	COAD	high	moderate	high	high	absent	moderate	moderate	absent	absent	low	moderate	high
Esophageal carcinoma	ESCA	low	low	high	moderate	absent	moderate	high	absent	absent	low	high	high
Liver hepatocellular carcinoma	LIHC	high	moderate	high	high	absent	moderate	moderate	absent	absent	low	high	high
Pancreatic adenocarcinoma	PAAD	moderate	moderate	high	high	low	moderate	high	absent	absent	moderate	high	moderate
Rectum adenocarcinoma	READ	high	moderate	high	high	absent	moderate	moderate	absent	absent	low	moderate	high
Stomach adenocarcinoma	STAD	high	moderate	high	moderate	absent	moderate	high	absent	absent	moderate	high	high
Gynecologic													
Breast invasive carcinoma	BRCA	absent	low	high	high	absent	moderate	high	absent	absent	low	high	high
Cervical and endocervical cancers	CESC	absent	low	high	high	absent	moderate	moderate	absent	absent	low	moderate	high
Ovarian serous cystadenocarcinoma	OV	absent	absent	high	high	low	moderate	high	absent	absent	moderate	high	high
Uterine corpus endometrial carcinoma	UCEC	absent	low	high	high	low	moderate	moderate	absent	absent	low	high	high
Head and neck													
Head and Neck squamous cell carcinoma	HNSC	absent	absent	high	high	absent	moderate	moderate	absent	absent	low	moderate	moderate
Hematologic and lymphatic malignancies													
Lymphoid neoplasm diffuse large B-cell lymphoma	DLBC	absent	absent	moderate	high	absent	moderate	moderate	absent	absent	low	moderate	moderate
Acute myeloid leukemia	LAML	absent	absent	high	high	absent	moderate	high	absent	absent	absent	absent	low
Thymoma	THYM	absent	absent	high	high	absent	moderate	moderate	absent	absent	low	moderate	moderate
Melanocytic													
Skin cutaneous melanoma	SKCM	absent	absent	high	high	moderate	moderate	high	absent	absent	low	high	moderate
Uveal melanoma	UVM	absent	absent	high	high	moderate	moderate	moderate	low	absent	low	moderate	high
Neural crest-derived													
Pheochromocytoma and Paraganglioma	PCPG	absent	absent	high	high	low	moderate	high	absent	absent	low	moderate	high
Soft tissue													
Sarcoma	SARC	absent	absent	high	high	low	moderate	moderate	absent	absent	moderate	high	moderate
Uterine carcinosarcoma	UCS	absent	low	high	high	low	moderate	moderate	absent	absent	moderate	high	high
Thoracic													
Lung adenocarcinoma	LUAD	absent	low	high	high	absent	moderate	high	absent	absent	moderate	high	high
Lung squamous cell carcinoma	LUSC	absent	low	high	high	absent	moderate	moderate	absent	absent	low	moderate	moderate
Mesothelioma	MESO	absent	absent	high	high	absent	moderate	moderate	absent	absent	moderate	high	high
Urologic													
Bladder urothelial carcinoma	BLCA	absent	low	high	high	absent	moderate	moderate	absent	absent	low	moderate	high
Kidney chromophobe	KICH	absent	absent	high	moderate	absent	moderate	high	absent	low	low	high	high
Kidney renal clear cell carcinoma	KIRC	moderate	moderate	high	high	absent	moderate	moderate	absent	absent	moderate	high	moderate
Kidney renal papillary cell carcinoma	KIRP	moderate	moderate	high	high	absent	moderate	moderate	absent	absent	low	high	high
Prostate adenocarcinoma	PRAD	absent	low	high	high	absent	moderate	moderate	absent	absent	moderate	high	high
Testicular Germ Cell Tumors	TGCT	low	absent	high	moderate	low	moderate	high	absent	absent	low	moderate	high

		Class III									Class IV			Class V		Class VI	Class 0	
**Disease name and pan-organ system**	**Cohort**	***ESR1***	***ESR2***	***ESRRA***	***ESRRB***	***ESRRG***	***NR3C1***	***NR3C2***	***PGR***	***AR***	***NR4A1***	***NR4A2***	***NR4A3***	***NR5A1***	***NR5A2***	***NR6A1***	***NR0B1***	***NR0B2***
Central nervous system (CNS)																		
Glioblastoma multiforme	GBM	low	low	moderate	absent	low	high	low	absent	low	moderate	low	low	absent	low	absent	absent	absent
Brain lower grade glioma	LGG	low	low	moderate	absent	low	high	moderate	low	low	moderate	low	low	absent	low	absent	low	absent
Endocrine																		
Adrenocortical carcinoma	ACC	low	low	high	absent	low	high	low	low	absent	high	low	moderate	high	low	absent	high	low
Thyroid carcinoma	THCA	moderate	absent	moderate	absent	low	high	moderate	low	low	high	moderate	low	absent	low	low	absent	absent
Gastrointestinal																		
Cholangiocarcinoma	CHOL	low	low	high	absent	low	high	low	low	low	high	low	low	absent	moderate	absent	absent	moderate
Colon adenocarcinoma	COAD	absent	absent	high	absent	low	low	moderate	absent	absent	high	low	low	absent	low	low	absent	low
Esophageal carcinoma	ESCA	low	low	high	absent	absent	high	low	low	absent	high	moderate	low	absent	low	low	absent	absent
Liver hepatocellular carcinoma	LIHC	low	absent	high	absent	absent	high	low	absent	low	moderate	low	low	absent	high	absent	absent	high
Pancreatic adenocarcinoma	PAAD	low	low	high	absent	low	high	moderate	low	low	high	moderate	moderate	absent	moderate	absent	absent	low
Rectum adenocarcinoma	READ	absent	absent	high	absent	low	low	low	absent	absent	high	low	low	absent	low	low	absent	low
Stomach adenocarcinoma	STAD	low	low	high	absent	low	moderate	moderate	low	low	high	moderate	moderate	absent	low	low	absent	low
Gynecologic																		
Breast invasive carcinoma	BRCA	high	absent	moderate	absent	low	high	low	moderate	moderate	high	moderate	low	absent	low	absent	absent	absent
Cervical and endocervical cancers	CESC	moderate	low	high	absent	absent	high	low	low	absent	high	moderate	low	absent	low	low	absent	absent
Ovarian serous cystadenocarcinoma	OV	high	low	moderate	absent	low	high	low	low	low	high	moderate	low	low	low	low	absent	absent
Uterine corpus endometrial carcinoma	UCEC	high	low	high	absent	low	low	low	high	low	high	low	low	absent	low	absent	absent	absent
Head and neck																		
Head and Neck squamous cell carcinoma	HNSC	low	low	high	absent	absent	high	low	absent	absent	high	low	low	absent	low	low	absent	absent
Hematologic and lymphatic malignancies																		
Lymphoid neoplasm diffuse large B-cell lymphoma	DLBC	low	low	high	absent	absent	high	low	absent	absent	moderate	low	low	absent	low	absent	absent	absent
Acute myeloid leukemia	LAML	low	low	moderate	absent	absent	high	low	absent	low	moderate	moderate	low	low	absent	low	absent	absent
Thymoma	THYM	low	low	high	absent	absent	high	low	absent	absent	moderate	low	low	low	low	absent	absent	absent
Melanocytic																		
Skin cutaneous melanoma	SKCM	low	low	high	absent	absent	high	low	absent	absent	high	moderate	low	absent	low	absent	absent	absent
Uveal melanoma	UVM	low	absent	high	absent	absent	moderate	low	absent	absent	high	low	high	absent	absent	absent	absent	absent
Neural crest-derived																		
Pheochromocytoma and Paraganglioma	PCPG	low	absent	moderate	absent	low	high	moderate	low	absent	high	moderate	moderate	absent	low	absent	absent	absent
Soft tissue																		
Sarcoma	SARC	low	absent	moderate	absent	absent	high	low	low	low	high	low	low	absent	low	absent	absent	absent
Uterine carcinosarcoma	UCS	low	low	high	absent	low	moderate	low	low	low	high	low	low	absent	low	absent	absent	absent
Thoracic																		
Lung adenocarcinoma	LUAD	low	low	high	absent	low	high	moderate	low	low	high	moderate	low	absent	low	low	absent	low
Lung squamous cell carcinoma	LUSC	low	low	high	absent	low	high	low	low	absent	high	moderate	low	absent	low	low	absent	absent
Mesothelioma	MESO	low	low	high	absent	absent	high	low	low	absent	high	moderate	low	absent	low	absent	absent	absent
Urologic																		
Bladder urothelial carcinoma	BLCA	low	low	high	absent	low	moderate	low	low	low	high	moderate	low	absent	low	absent	absent	absent
Kidney chromophobe	KICH	low	absent	high	low	high	high	high	moderate	low	high	moderate	low	absent	low	absent	absent	high
Kidney renal clear cell carcinoma	KIRC	low	low	high	absent	low	high	moderate	low	low	high	moderate	moderate	absent	low	absent	absent	absent
Kidney renal papillary cell carcinoma	KIRP	low	low	high	absent	low	high	moderate	absent	low	moderate	low	low	absent	low	absent	absent	absent
Prostate adenocarcinoma	PRAD	low	low	high	absent	low	moderate	low	low	moderate	high	moderate	low	absent	low	absent	absent	absent
Testicular Germ Cell Tumors	TGCT	low	low	moderate	low	low	moderate	low	absent	absent	high	low	low	low	low	low	low	absent

Note: Expression levels (log10-transformed) were stratified according to quartiles, where Q1 (0-25%: -Inf to 0.98) were defined as absent, Q2 (25-50%: 0.98 to 2.32) as low, Q3 (50-75%: 2.32 to 2.93) as moderate, and Q4 (75-100%: 2.93 to 5.13) as high expression.

Supplementary Table 2. Correlation matrix for NR gene pairs in TCGA cancer types with -0.6 < *r* > 0.6 (strong correlation)

AR	ESR1	ESR2	ESRRA	ESRRB	ESRRG	HNF4A	HNF4G	NR0B1	NR0B2	NR1D1	NR1D2	NR1H2	NR1H3	NR1H4	NR1I2	NR1I3	NR2C1	NR2C2	NR2E1	NR2E3	NR2F1	NR2F2	NR2F6	NR3C1	NR3C2	NR4A1	NR4A2	NR4A3	NR5A1	NR5A2	NR6A1	PGR	PPARA	PPARD	PPARG	RARA	RARB	RARG	RORA	RORB	RORC	RXRA	RXRB	RXRG	THRA	THRB	VDR
AR	●											●						●●			●		●●	●●								●●	●						●	●							
	ESR1							●													●			●●								●●●							●	●●				●			
		ESR2											●										●						●												●						
			ESRRA									●											●	●																							
				ESRRB																																								●			
					ESRRG				●	●				●											●					●											●						
						HNF4A	●●●		●						●									●						●					●			●			●						
							HNF4G		●						●							●		●	●					●					●			●			●						
								NR0B1																								●												●			
									NR0B2						●										●					●					●			●			●●●						
										NR1D1																																					
											NR1D2							●				●			●●											●	●		●●							●●	
												NR1H2						●●					●●●	●																							●
													NR1H3																																		
														NR1H4																●																	
															NR1I2										●					●					●												
																NR1I3		●																													
																	NR2C1																														
																		NR2C2					●	●	●								●●				●		●●								
																			NR2E1																												
																				NR2E3			●																								
																					NR2F1											●●					●	●									
																						NR2F2			●					●						●	●	●							●		
																							NR2F6	●●									●						●●	●							
																								NR3C1								●●●					●●		●●	●							
																									NR3C2								●	●	●		●		●		●●					●●	
																										NR4A1	●●●●●●	●●●●●●																			
																											NR4A2	●																			
																												NR4A3																			
																													NR5A1								●				●						
																														NR5A2					●			●									
																															NR6A1																
																																PGR					●		●	●●				●			
																																	PPARA														
																																		PPARD		●			●			●				●	
																																			PPARG												
																																				RARA		●●	●			●			●●●	●	
																																					RARB	●●			●					●	
																																						RARG			●	●					
																																							RORA			●				●	
																																								RORB							
																																									RORC						
																																										RXRA					
																																											RXRB				
																																												RXRG			
																																													THRA	●	
																																														THRB	
																																															VDR

Note: Each cancer type is denoted with a colored ● symbol (● ESCA, ● KIHC, ● PAAD, ● PRAD, ● STAD, ● THYM).
